# E3 Ligase Rbx1 Orchestrates Thymus Development and Fate Determination of αβ-γδ T Cells

**DOI:** 10.34133/research.0774

**Published:** 2025-07-10

**Authors:** Di Wu, Qiuxu Chen, Mingjia Tan, Yi Sun

**Affiliations:** ^1^ Cancer Institute (Key Laboratory of Cancer Prevention and Intervention, China National Ministry of Education) of the Second Affiliated Hospital, and Institute of Translational Medicine, Zhejiang University School of Medicine, Hangzhou 310029, China.; ^2^ Cancer Center of Zhejiang University, Hangzhou 310029, China.; ^3^ Research Center for Life Science and Human Health, Binjiang Institute of Zhejiang University, Hangzhou 310053, China.; ^4^ Department of Radiation Oncology, University of Michigan, Ann Arbor, MI 48109, USA.; ^5^ State Key Laboratory of Transvascular Implantation Devices, the Second Affiliated Hospital, Zhejiang University School of Medicine, Hangzhou 310029, China.

## Abstract

T lymphocytes consist of αβ and γδ T cells, which mature and differentiate from the same progenitor cells in the thymus. Cullin-RING ligases (CRLs), the largest family of ubiquitin ligases, require neddylation on the scaffold protein Cullins for their ligase activity. The role of neddylation–CRL system in thymus development and fate determination of αβ/γδ T cells remains elusive. Here, we generated conditional knockout mouse models with thymus individual deletion of Ube2m or Ube2f, 2 neddylation E2-conjugating enzymes, and Rbx1 or Sag, 2 dual neddylation and ubiquitylation E3 ligases. We found that only Rbx1, but not Ube2m/Ube2f, nor Sag, plays an essential role in thymus development and fate determination of αβ/γδ T cells. Specifically, Rbx1 loss causes shrinkage of the thymus, delayed T cell development, increased γδ T cells in the thymus, increased the ratio of immature *Gzma*^+^ γδ T cells, and decreased the ratio of the proliferative subpopulation. Some of these phenotypes were moderately rescued by simultaneous Bim deletion. Mechanistically, Rbx1 loss alters the Akt, NF-κB, and metabolic pathways in progenitor γδ T cells/DN3a cells. Finally, *Rbx1* loss altered the γδ T1/T17 cell population in the thymus, suggesting that Rbx1 controls the fate of γδ T cells.

## Introduction

The thymus is the site at which T lymphocytes develop and mature from precursors migrating from the bone marrow. T lymphocytes pass through several stages and checkpoints in the thymus, from CD4^−^CD8^−^ double negative (DN) cells to CD4^+^CD8^+^ double positive (DP) cells, and finally to CD4^+^ or CD8^+^ single positive (SP) cells. The DN stage is the initial stage of T cell maturation and can itself be divided into 4 steps: CD25^−^CD44^+^ (DN1), CD25^+^CD44^+^ (DN2), CD25^+^CD44^−^ (DN3), and CD25^−^CD44^−^ (DN4). The detailed developmental processes and underlying mechanisms are important research topics in immunology [1].

The diversity of T cell receptors (TCRs) classifies T lymphocytes into 2 subtypes: αβ and γδ, which are fundamentally distinct lineages. The αβ T lymphocytes possess TCRs comprising α and β chains; these cells include CD4^+^ helper T cells and CD8^+^ cytotoxic T cells in the periphery. In contrast, the TCRs of γδ T lymphocytes comprise γ and δ chains; these cells play a role in innate immunity [2], with a marked role against infection [3,4] and tumor development [5]. The αβ and γδ T cells differentiate from the same progenitor in the thymus at the DN3 stage, while αβ T cells are further developed to the DP stage [6]. The mechanism that determines the differentiation of T cells to αβ or γδ subtypes remains elusive [7]. With regard to cytokine production, γδ T cells can be further classified into interferon-γ (IFN-γ)-producing (γδ T1) cells or interleukin-17 (IL-17)-secreting (γδ T17) cells, each of which have different functions [8].

Cullin-RING ligase (CRL), the largest member of the ubiquitin E3 ligase family, uses RING-box 1 (Rbx1/Roc1) or RING-box 2 (Rbx2/Rnf7/Sag) as the catalytic subunit [9,10]. The ubiquitin ligase activity of CRLs requires neddylation, a ubiquitylation-like modification, on the scaffold Cullin protein. In the neddylation cascade, Ube2m and Ube2f act as E2 NEDD8-conjugating enzymes, whereas Rbx1 and Sag act as E3 NEDD8 ligases. Ube2m couples Rbx1, and Ube2f couples Sag, to catalyze neddylation of cullins 1 to 4 or cullin 5 to activate CRL1 to CRL4 or CRL5, respectively [11]. Previous studies showed that the Ube2m–Rbx1 and Ube2f–Sag axes are functionally nonredundant [12,13]. The neddylation–CRL system regulates multiple biological processes [14–16], including the immune system [17,18], particularly the functional regulation of regulatory T cells (Treg cells) [19–21], T lymphocytes [22,23], dendritic cells [24,25], neutrophils [26], and macrophages [27,28]. However, whether and how the neddylation–CRL system functions during thymus development and fate determination of αβ/γδ T cells remains unclear.

Lck kinase is expressed by the earliest thymic immigrants and by all T cell subsets [29]. Therefore, the *Lck^Cre^-Loxp* system has been used to knock out various genes in cells at an early stage of thymus development [30,31]. Here, we used the *Lck^Cre^*-*Loxp* system to conditionally knock out 4 genes, including *Ube2m*, *Ube2f* (neddylation E2s) and *Rbx1*, *Sag* (dual E3s for neddylation and CRL ubiquitylation) in early DN cells in the thymus. The goal is to determine the role of the neddylation–CRL system in thymus development and fate determination of αβ/γδ T cells. We report here that while the thymus-specific deletion of *Sag*, *Ube2f* or *Ube2m* in mice did not have obvious phenotypic changes, indicating neither of these genes plays any significant detectable role in the thymus development, *Rbx1* deletion causes remarkable phenotypes. Specifically, *Rbx1* loss led to shrinkage of the thymus, with delayed development of T cells, and elevated γδ T cells. Although *Rbx1* deletion caused the accumulation of Bim substrate, a pro-apoptosis protein [32], simultaneous Bim deletion only showed minor phenotype rescue, indicating that Bim does not play a major role. The mechanistic study using bulk RNA sequencing of DN3a cells (progenitor γδ T cells in the thymus) revealed that *Rbx1* regulates Akt, nuclear factor κB (NF-κB), and metabolic pathways. Single-cell RNA sequencing of thymus γδ T cells revealed that *Rbx1* deficiency caused an increase in the *Gzma*^+^ immature γδ T cell and a decrease in the proliferative immature γδ T cell subpopulation in the thymus . *Rbx1* deficiency also altered the ratio of γδ T1/T17 cells in the thymus, suggesting that Rbx1 determines the fate of γδ T1/T17 cells and function of mature γδ T cells.

## Results

### The Ube2f–Sag axis has no effect on thymus development or fate determination of αβ/γδ T cells

**Fig. 1.**
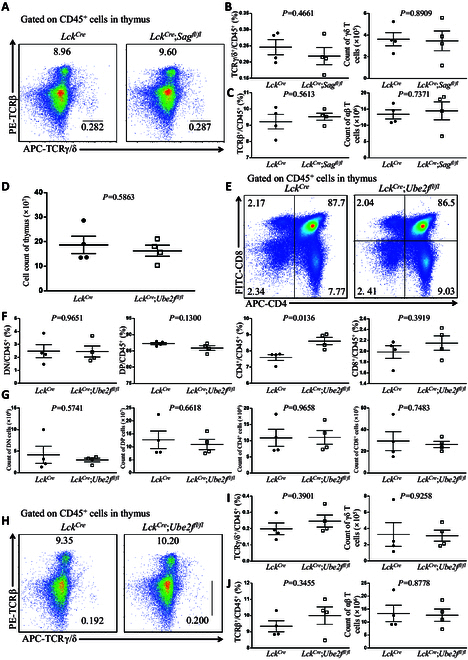
The Ube2f–Sag axis does not play an obvious role in thymus development and γδ T cell generation. (A) FACS analysis of TCRβ and TCRγ/δ on CD45^+^ cells from thymus in *Lck^Cre^* and *Lck^Cre^*;*Sag*^*fl*/*fl*^ mice at 8 weeks old. (B) Ratios and numbers of γδ T cells in the thymus from *Lck^Cre^* and *Lck^Cre^*;*Sag*^*fl*/*fl*^ mice at 8 weeks old (*n* = 4). (C) Ratios and numbers of αβ T cells in the thymus from *Lck^Cre^* and *Lck^Cre^*;*Sag*^*fl*/*fl*^ mice at 8 weeks old (*n* = 4). (D) Cell numbers of the thymus from *Lck^Cre^* and *Lck^Cre^*;*Ube2f*^*fl*/*fl*^ mice at 8 weeks old (*n* = 4). (E) FACS analysis of CD4 and CD8 on CD45^+^ cells from thymus in *Lck^Cre^* and *Lck^Cre^*;*Ube2f*^*fl*/*fl*^ mice at 8 weeks old. (F) Ratios of DN, DP, SP1, and SP2 cells among CD45^+^ cells in the thymus from *Lck^Cre^* and *Lck^Cre^*;*Ube2f*^*fl*/*fl*^ mice at 8 weeks old (*n* = 4). (G) Cell numbers of DN, DP, SP1, and SP2 cells among CD45^+^ cells in the thymus from *Lck^Cre^* and *Lck^Cre^*;*Ube2f*^*fl*/*fl*^ mice at 8 weeks old (*n* = 4). (H) FACS analysis of TCRβ and TCRγ/δ on CD45^+^ cells from thymus in *Lck^Cre^* and *Lck^Cre^*;*Ube2f*^*fl*/*fl*^ mice at 8 weeks old. (I) Ratios and numbers of γδ T cells in the thymus from *Lck^Cre^* and *Lck^Cre^*;*Ube2f*^*fl*/*fl*^ mice at 8 weeks old (*n* = 4). (J) Ratios and numbers of αβ T cells in the thymus from *Lck^Cre^* and *Lck^Cre^*;*Ube2f*^*fl*/*fl*^ mice at 8 weeks old (*n* = 4).

We cloned the *Sag* gene in 1999 [10] and have previously reported that in *Lck^Cre^*;*Sag*^*fl*/*fl*^ mice, *Sag* loss does not affect thymus development or the ratios and numbers of populations of DN, DP, CD4^+^, or CD8^+^ cells in the thymus [22]. Here, we asked whether Sag affects the generation of γδ T cells in the thymus. In *Lck^Cre^*;*Sag*^*fl*/*fl*^ mice lacking *Sag* expression in the thymus (Fig. S1A), we found that neither the ratio nor the absolute number of γδ T cells and αβ T cells was different from those in *Lck^Cre^* control mice (Fig. 1A to C), indicating that Sag is not involved in γδ T cell generation in the thymus.

We further investigated the role of Ube2f, a neddylation E2 paring with Sag E3 for cullin 5 neddylation [11], in the thymus by generating *Lck^Cre^*;*Ube2f*^*fl*/*fl*^ mice lacking *Ube2f* in the thymus (Fig. S1B). The results showed that compared to *Lck^Cre^* control mice, the total cell number in the thymus of *Lck^Cre^*;*Ube2f*^*fl*/*fl*^ mice remained largely unchanged (Fig. 1D), and the ratios of DN, DP, SP1, and SP2 cells to total CD45^+^ cells were also similar, despite a slight increase in the CD4^+^ to CD45^+^ cell ratio (Fig. 1E and F). Consistently, the numbers of DN, DP, SP1, and SP2 cells in *Lck^Cre^*;*Ube2f*^*fl*/*fl*^ mice were not significantly different from those in *Lck^Cre^* control mice (Fig. 1G). Finally, the *Ube2f* loss had no effect on the ratio and number of both γδ T cells and αβ T cells in the thymus, indicating a lack of effect on γδ T cell generation (Fig. 1H to J). Collectively, Ube2f does not play any obvious role in regulating thymus development or fate determination of αβ/γδ T cells.

### Ube2m has no effect on thymus development and minimal, if any, on fate determination αβ-γδ T cells

**Fig. 2.**
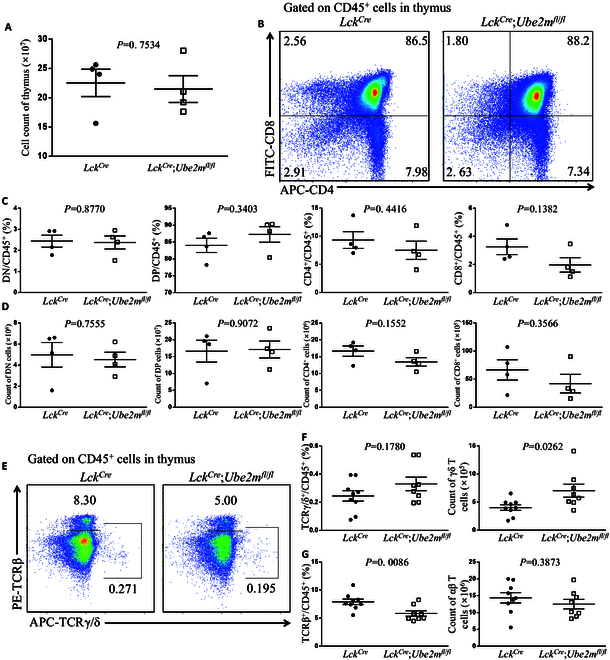
Analysis of the thymus from *Lck^Cre^*;*Ube2m*^*fl*/*fl*^ mice. (A) Cell numbers of the thymus from *Lck^Cre^* and *Lck^Cre^*;*Ube2m*^*fl*/*fl*^ mice at 8 weeks old (*n* = 4). (B) FACS analysis of CD4 and CD8 on CD45^+^ cells from thymus in *Lck^Cre^* and *Lck^Cre^*;*Ube2m*^*fl*/*fl*^ mice at 8 weeks old. (C) Ratios of DN, DP, SP1, and SP2 cells among CD45^+^ cells in the thymus from *Lck^Cre^* and *Lck^Cre^*;*Ube2m*^*fl*/*fl*^ mice at 8 weeks old (*n* = 4). (D) Cell numbers of DN, DP, SP1, and SP2 cells in the thymus from *Lck^Cre^* and *Lck^Cre^*; *Ube2m*^*fl*/*fl*^ mice at 8 weeks old (*n* = 4). (E) FACS analysis of TCRβ and TCRγ/δ on CD45^+^ cells from thymus in *Lck^Cre^* and *Lck^Cre^*;*Ube2m*^*fl*/*fl*^ mice at 8 weeks old. (F) Ratios and numbers of γδ T cells in the thymus from *Lck^Cre^* and *Lck^Cre^*;*Ube2m*^*fl*/*fl*^ mice at 8 weeks old (*n* = 8 to 9). (G) Ratios and numbers of αβ T cells in the thymus from *Lck^Cre^* and *Lck^Cre^*;*Ube2m*^*fl*/*fl*^ mice at 8 weeks old (*n* = 8 to 9).

We next determined the possible effect of Ube2m, a neddylation E2-conjugating enzyme, coupling with downstream Rbx1 E3 for neddylation of cullins 1 to 4 [11], in thymus development. To this end, we generated *Lck^Cre^*;*Ube2m*^*fl*/*fl*^ mice lacking expression of the *Ube2m* gene in the thymus (Fig. S1C). Consistent with the observations made in *Lck^Cre^*;*Ube2f*^*fl*/*fl*^ mice, the development of the thymus in *Lck^Cre^*;*Ube2m*^*fl*/*fl*^ mice was little affected. Specifically, the total cell numbers (Fig. 2A), and the percentages and absolute numbers of DN, DP, and SPs cells, did not change significantly upon deletion of *Ube2m* (Fig. 2B to D). The percentage of γδ T cells in the thymus of *Lck^Cre^*;*Ube2m*^*fl*/*fl*^ mice was similar to that in control mice. However, while the ratio of TCRγ/δ^+^ cells had no difference, the number of γδ T cells was significantly increased (Fig. 2E and F). On the other hand, while the number of αβ T cells had no change, the ratio of TCRβ^+^ cells was significantly reduced (Fig. 2E and G). We concluded that Ube2m does not play a role in regulating thymus development and might have a minor, if any, effect on γδ T cell generation.

### Rbx1 regulates thymus development and γδ T cell generation

**Fig. 3.**
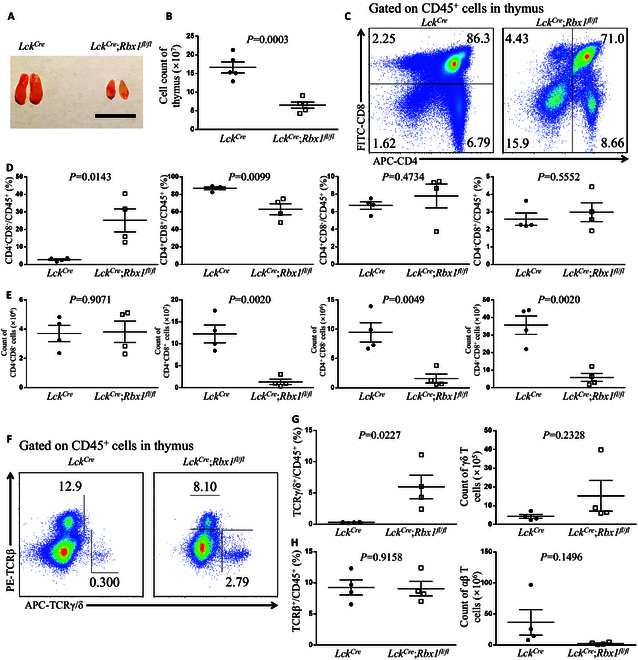
Rbx1 regulates thymus development and γδ T cell generation. (A) Image of the thymus from *Lck^Cre^* and *Lck^Cre^*;*Rbx1*^*fl*/*fl*^ mice at 8 weeks old (scale bar, 1 cm). (B) Cell numbers of the thymus from *Lck^Cre^* and *Lck^Cre^*;*Rbx1*^*fl*/*fl*^ mice at 8 weeks old (*n* = 5). (C) FACS analysis of CD4 and CD8 on CD45^+^ cells from thymus in *Lck^Cre^* and *Lck^Cre^*;*Rbx1*^*fl*/*fl*^ mice at 8 weeks old. (D) Ratios of DN, DP, SP1, and SP2 cells among CD45^+^ cells in the thymus from *Lck^Cre^* and *Lck^Cre^*;*Rbx1*^*fl*/*fl*^ mice at 8 weeks old (*n* = 4). (E) Cell numbers of DN, DP, SP1, and SP2 cells in the thymus from *Lck^Cre^* and *Lck^Cre^*;*Rbx1*^*fl*/*fl*^ mice at 8 weeks old (*n* = 4). (F) FACS analysis of TCRβ and TCRγ/δ on CD45^+^ cells from thymus in *Lck^Cre^* and *Lck^Cre^*;*Rbx1*^*fl*/*fl*^ mice at 8 weeks old. (G) Ratios and numbers of γδ T cells in the thymus from *Lck^Cre^* and *Lck^Cre^*;*Rbx1*^*fl*/*fl*^ mice at 8 weeks old (*n* = 4). (H) Ratios and numbers of αβ T cells in the thymus from *Lck^Cre^* and *Lck^Cre^*;*Rbx1*^*fl*/*fl*^ mice at 8 weeks old (*n* = 4).

Next, we explored the function of Rbx1, a dual E3 ligase for neddylation, which couples with Ube2m E2 for neddylation of cullins 1 to 4 [11], and the catalytic subunit of CRL1 to CRL4, in the thymus by generating *Lck^Cre^*;*Rbx1*^*fl*/*fl*^ conditional knockout mice (Fig. S1D). Notably, the size of the thymus in *Lck^Cre^*;*Rbx1*^*fl*/*fl*^ mice was clearly smaller than that in control mice (Fig. 3A and Fig. S1E and F). Consistently, the cellularity of the thymus in *Lck^Cre^*;*Rbx1*^*fl*/*fl*^ mice was only one-third of the normal level (Fig. 3B). These results indicated that *Rbx1* deficiency has a marked effect on the development of the thymus.

We next determined the role of Rbx1 in the T cell development in the thymus and found that the ratio of DN cells was higher in *Lck^Cre^*;*Rbx1*^*fl*/*fl*^ mice (Fig. 3C and left panel of Fig. 3D), despite that the counting of DN cells was not altered in *Lck^Cre^*;*Rbx1*^*fl*/*fl*^ mice (left panel of Fig. 3E) due to shrinking of the *Rbx1* deleted thymus (Fig. 3B). The ratio of DP cells was decreased, and ratios of SP cells were not significantly altered in *Lck^Cre^*;*Rbx1*^*fl*/*fl*^ mice (Fig. 3D), while the absolute cell number of DP and SPs was all decreased dramatically (Fig. 3E) due to the decrease of cellularity of the thymus (Fig. 3B) in *Lck^Cre^*;*Rbx1*^*fl*/*fl*^ mice. All these findings suggest that *Rbx1* deficiency inhibits T cell development, and Rbx1 regulation of thymus development is not simply affecting cell proliferation and survival.

We further investigated whether Rbx1 affects the generation of γδ T cells in the thymus. Notably, the ratio of γδ T cells to CD45^+^ immune cells was significantly elevated (Fig. 3F and G), while the absolute number of γδ T cells was elevated in the thymus of *Lck^Cre^*;*Rbx1*^*fl*/*fl*^ with no significance (Fig. 3G), despite an obvious reduction in the total cell number in the thymus (Fig. 3B). Deletion of *Rbx1* caused an obvious increase in the number and ratio of γδ T cells in the thymus, suggesting that Rbx1 plays a pivotal role in determining the fate of αβ/γδ T cells. Given the fact that deletion of upstream *Ube2m* E2 in the thymus has no dramatic phenotype, the observed thymus effect of *Rbx1* deletion is likely attributable to its CRL E3 ligase activity, not its E3 neddylation activity.

### Bim plays a minor role in Rbx1 regulation of the thymus development

**Fig. 4.**
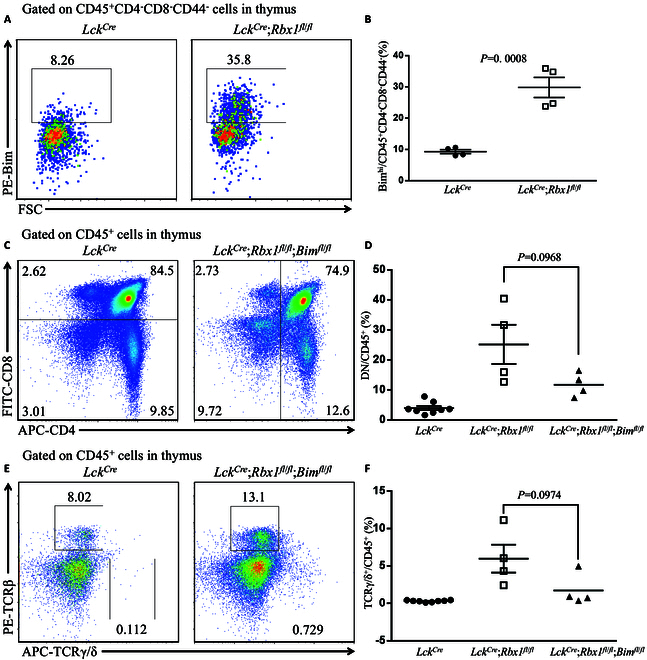
Research about the role of Bim in function of Rbx1 in the thymus. (A) FACS analysis of Bim in CD45^+^CD4^−^CD8^−^CD44^−^ cells from thymus in *Lck^Cre^* and *Lck^Cre^*;*Rbx1*^*fl*/*fl*^;*Bim*^*fl*/*fl*^ mice at 8 weeks old. (B) Ratios of Bim^hi^ cells among DN3&4 cells in the thymus from *Lck^Cre^*, *Lck^Cre^*;*Rbx1*^*fl*/*fl*^ and *Lck^Cre^*;*Rbx1*^*fl*/*fl*^;*Bim*^*fl*/*fl*^ mice at 8 weeks old (*n* = 4). (C) FACS analysis of CD4 and CD8 on CD45^+^ cells from thymus in *Lck^Cre^* and *Lck^Cre^*;*Rbx1*^*fl*/*fl*^;*Bim*^*fl*/*fl*^ mice at 8 weeks old. (D) Ratios of DN cells among CD45^+^ cells in the thymus from *Lck^Cre^*, *Lck^Cre^*;*Rbx1*^*fl*/*fl*^ and *Lck^Cre^*;*Rbx1*^*fl*/*fl*^;*Bim*^*fl*/*fl*^ mice at 8 weeks old (*n* = 4 to 8). (E) FACS analysis of TCRβ and TCRγ/δ on CD45^+^ cells from thymus in *Lck^Cre^* and *Lck^Cre^*;*Rbx1*^*fl*/*fl*^;*Bim*^*fl*/*fl*^ mice at 8 weeks old. (F) Ratios of γδ T cells among CD45^+^ cells in the thymus from *Lck^Cre^*, *Lck^Cre^*;*Rbx1*^*fl*/*fl*^ and *Lck^Cre^*;*Rbx1*^*fl*/*fl*^;*Bim*^*fl*/*fl*^ mice at 8 weeks old (*n* = 4 to 8).

We next investigated possible mechanism by which Rbx1 controls thymus development by focusing on pro-apoptotic protein Bim, a Rbx1 substrate (*34*), known to be involved in immune regulation [33]. The observation that *Rbx1* deletion significantly reduced the thymus size (Fig. 3A and B and Fig. S1F) could imply the apoptosis induced by accumulated Bim upon *Rbx1* deletion. We first confirmed that Bim protein is indeed accumulated in DN3&4 cells from the thymus of *Lck^Cre^*;*Rbx1*^*fl*/*fl*^ mice (Fig. 4A and B). We then generated *Lck^Cre^*;*Rbx1*^*fl*/*f*^;*Bim1*^*fl*/*fl*^ mice lacking both *Rbx1* and *Bim* in the thymus for a rescue experiment. However, similar to the observation made in *Lck^Cre^*;*Rbx1*^*fl*/*fl*^ mice (Fig. 3), the thymus of double knockout mice was also shown to have reduced size and cellularity (Fig. S2A and B), which is similar with those in *Lck^Cre^*;*Rbx1*^*fl*/*fl*^ mice (Fig. S2C and D). The ratio of DN cells to total CD45^+^ cells (Fig. 4C and Fig. S2E and F), as well as the ratio of γδ T cells to total CD45^+^ cells (Fig. 4E and Fig. S2G and H), were also elevated. However, the elevated ratio of DN cells to total CD45^+^ cells (Fig. 4D) as well as of γδ T cells to total CD45^+^ cells (Fig. 4F) in the thymus were to a lesser extent in *Lck^Cre^*;*Rbx1*^*fl*/*f*^;*Bim1*^*fl*/*fl*^ double-null mice than in *Lck^Cre^*;*Rbx1*^*fl*/*fl*^ mice, although the differences were not statistically significant. Taken together, Bim plays a moderate, if any, role in defective development of the thymus caused by *Rbx1* deficiency.

### Rbx1 regulates differentiation of the DN3 and DN4 cell populations

**Fig. 5.**
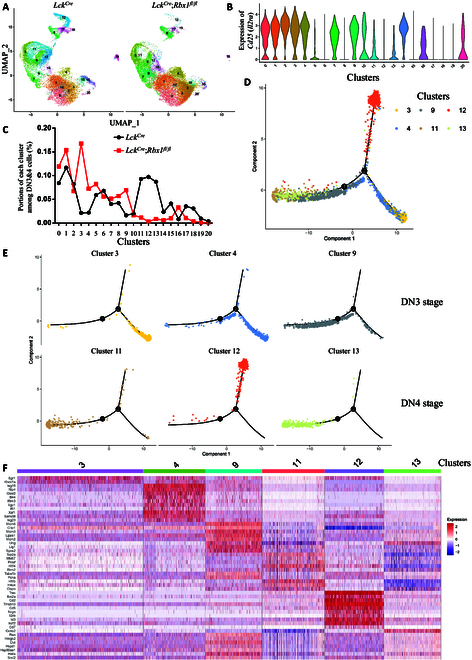
Single-cell RNA sequencing research about Rbx1 function on DN3 and DN4 cells. (A) UMAP visualization of single-cell transcriptomics data of DN3 and DN4 cells in the thymus from *Lck^Cre^* and *Lck^Cre^*;*Rbx1*^*fl*/*fl*^ mice. (B) Expression of *Il2ra* gene in each cluster of DN3 and DN4 cells in the thymus from *Lck^Cre^* and *Lck^Cre^*;*Rbx1*^*fl*/*fl*^ mice. (C) Proportions of each cluster among DN3 and DN4 cells in the thymus from *Lck^Cre^* and *Lck^Cre^*;*Rbx1*^*fl*/*fl*^ mice. (D and E) Pseudotime analysis of clusters 3, 4, 9, 11, 12, and 13 from DN3 and DN4 cells in the thymus from *Lck^Cre^* and *Lck^Cre^*;*Rbx1*^*fl*/*fl*^ mice. (F) Top 10 marker genes of clusters 3, 4, 9, 11, 12, and 13 of DN3 and DN4 cells in the thymus from *Lck^Cre^* and *Lck^Cre^*;*Rbx1*^*fl*/*fl*^ mice.

Next, we examined the effect of *Rbx1* deletion in the subpopulations of DN3 and DN4 (referred as DN3&4) cells at the later stages of DN cells in the thymus development at the molecular levels. The DN3&4 cells (CD45^+^CD4^−^CD8^−^CD44^−^) were first sorted from the thymus of *Lck^Cre^* control and *Lck^Cre^*;*Rbx1*^*fl*/*fl*^ mice by fluorescence-activated cell sorting (FACS) and subjected to single-cell RNA sequencing. The Uniform Manifold Approximation and Projection (UMAP) result showed that DN3&4 cells can be divided into several clusters (Fig. 5A). As expected, *CD45* (*Ptprc*) was expressed at high levels by all subpopulations, whereas *CD4-*, *CD8a-*, and *CD44*-expressing cells were barely detectable within the DN3&4 cell population (Fig. S3). The high expression of *CD25* (*Il2ra*) in clusters 0 to 4, 7, 9, and 14 (Fig. 5B and Fig. S3) suggested that these clusters correspond to DN3 cells (i.e., CD25^+^CD44^−^), whereas very low expression of *CD25* (*Il2ra*) in clusters 5, 6, 8, 10 to 13, and 15 to 20 (Fig. 5B and Fig. S3) suggested that these clusters correspond to DN4 cells (i.e., CD25^−^CD44^−^). In the *Rbx1*-deficient thymus, the percentage of DN3 cells increased from 40.56% to 70.63%, while that of DN4 cells decreased from 59.44% to 29.37%, indicating that *Rbx1* deletion prevents the differentiation of DN3 to DN4 cells in vivo.

When each cluster was compared, *Rbx1* deletion caused a marked increase in clusters 3, 4, and 9, and a marked reduction in clusters 11 to 13, in the DN3&4 cell population (Fig. 5C). To examine the developmental trajectory, we performed a pseudo-time analysis on the above clusters and identified 4 developmental stages (Fig. 5D and Fig. S4). As shown in Fig. 5E, clusters 3 and 4 within the DN3&4 cell population were developed and divided into 2 branches: clusters 9, 11, and 13 were located in one branch, while cluster 12 was located in another branch. Upon *Rbx1* deletion, clusters 3, 4, and 9 could not move forward to clusters 11 to 13, indicating that *Rbx1* deficiency delays the developmental process.

To further explore the characteristics of these clusters, we analyzed expression of marker genes and associated signaling pathways (Tables S1 and S2), focusing on clusters 3, 4, 9 11, 12, and 13 (Fig. 5F and Fig. S5), the proportions of which were altered most significantly upon *Rbx1* deletion. Among the different marker genes (Fig. 5F), we found a list of the following genes, including *Egr1*, which plays a role at multiple stages of thymocyte development [34]; *Isg15*, which is up-regulated in the thymus during early pregnancy [35]; *TOP2A*, which is expressed at high levels in the thymus of patients with Down syndrome [36]; *Rrm2*, a gene down-regulated in mouse thymoma cells treated with tributyltin oxide [37]; *Itm2a*, a important regulator of thymus development [38]; *Cd2*, which plays pivotal function during fetal thymus ontogeny [39]; *Cd5*, a gene involved in negative selection in the thymus [40]; *Il2rb*, which plays a role in regulating normal lymphocyte development in vivo [41]; *Id3*, which acts in a transient fashion downstream of extracellular signals such as TCR signaling and is involved in thymus development [42]; *Ran*, a gene expressed in various organs and tissues, including the thymus cortex [43]; and *Hmgb2*, which is abundant in the thymus [44]. Notably, among the above genes, *Egr1* is abundant in cluster 3, *Isg15* is abundant in cluster 4, *TOP2A* and *Rrm2* are abundant in cluster 11, *Itm2a*, *Cd2*, *Cd5*, *Il2rb*, and *Id3* are abundant in cluster 12, and *Ran* and *Hmgb2* are abundant in cluster 13 (Fig. 5F). We further revealed how these marker genes vary in the corresponding cell subpopulations in the thymus by showing the expression of selected marker genes via UMAP pattern of DN3&4 cells in the thymus (Fig. S6). The function of few other marker genes may also be related to thymus development, an interesting project for future investigation.

### Analysis of transcriptomic alterations in *Rbx1*-deficient DN3a cells in the thymus

**Fig. 6.**
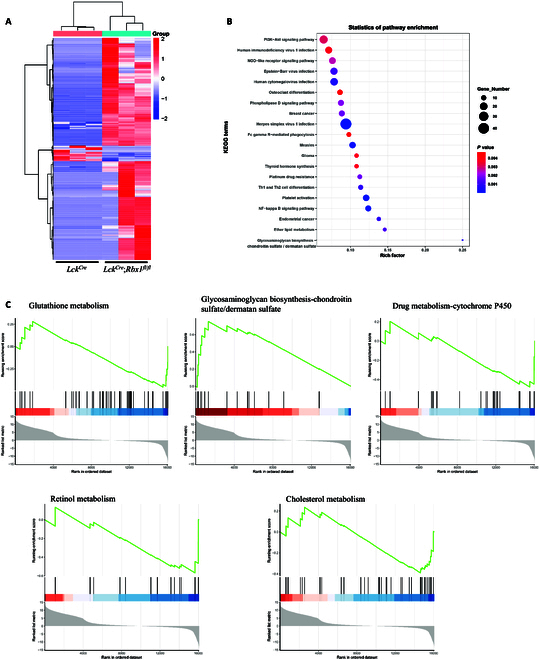
Transcriptional analyses of *Rbx1*-deficient DN3a cells in the thymus. (A) Unsupervised cluster analysis of the transcriptional data in *Rbx1*-deficient DN3a cells with ∣log_2_ fold change∣ > 1 and *P* < 0.05. (B) KEGG pathway analysis of the transcriptional data in *Rbx1*-deficient DN3a cells with ∣log_2_ fold change∣ > 1 and *P* < 0.05. (C) Selected results of GSEA pathway analysis of the transcriptional data in *Rbx1*-deficient DN3a cells.

It is well known that αβ and γδ T cells differentiate from the same progenitor cells [45]. At the DN3 stage, progenitor cells can be divided into DN3a and DN3b cells: DN3a cells develop into γδ T cells, while DN3b cells develop into αβ T cells [45]. DN3a cells are characterized by low expression of the CD28 protein [46]. Therefore, to investigate how *Rbx1* affects generation of γδ T cells, we examined transcriptomic alterations in *Rbx1*-deficient DN3a cells. DN3a cells (CD4^−^CD8^−^CD25^hi^CD44^−^CD28^−^) [47] were isolated from the thymus of *Lck^Cre^* and *Lck^Cre^*;*Rbx1*^*fl*/*fl*^ mice by FACS and subjected to RNA sequencing. Unsupervised cluster analysis of the transcriptional data showing a >2-fold or <0.5-fold change in expression with a *P* value of <0.05 (i.e., ∣log_2_ fold change∣ > 1 and *P* < 0.05) revealed that *Rbx1* deletion caused marked alterations in the transcriptome of DN3a cells (Fig. 6A).

To explore this further, we conducted Kyoto Encyclopedia of Genes and Genomes (KEGG) analysis to identify genes with ∣log_2_ fold change∣ > 1 and *P* < 0.05. As shown in Fig. 6B, deleting *Rbx1* caused robust changes in genes belonging to multiple pathways. Among them, Akt [48] and NF-κB [49] are known to regulate the development of the thymus (Fig. 6B and Fig. S7).

To achieve a more comprehensive understanding of the mechanism by which Rbx1 operates in DN3a cells, we performed Gene Set Enrichment Analysis (GSEA) of the pathways affected by *Rbx1* deficiency. The metabolic pathways identified included glutathione metabolism, glycosaminoglycan biosynthesis, drug metabolism, retinol metabolism, and cholesterol metabolism (Fig. 6C and Fig. S8). Taken together, these data demonstrated that Rbx1 regulates the transcriptome of many signaling pathways in DN3a cells, particularly the Akt, NF-κB, and metabolic pathways in vivo.

### The function of Rbx1 in γδ T cells in the thymus

**Fig. 7.**
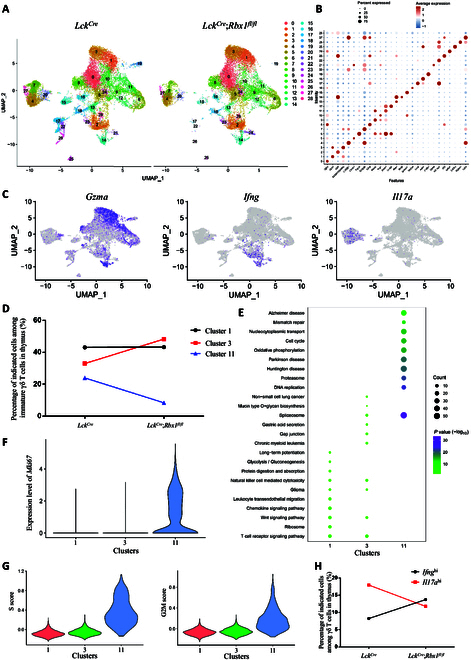
Subpopulation analysis of *Rbx1*-deficient γδ T cells in the thymus. (A) UMAP visualization of single-cell transcriptomics data of γδ T cells in the thymus from *Lck^Cre^* and *Lck^Cre^*;*Rbx1*^*fl*/*fl*^ mice. (B) Top 1 marker genes of each cluster of γδ T cells in the thymus from *Lck^Cre^* and *Lck^Cre^*;*Rbx1*^*fl*/*fl*^ mice. (C) Expression of *Gzma*, *Ifng*, and *Il17a* genes in each cluster of γδ T cells in the thymus from *Lck^Cre^* and *Lck^Cre^*;*Rbx1*^*fl*/*fl*^ mice. (D) Ratios of cluster 1, 3, and 11 cells among thymus immature γδ T cells in the thymus from *Lck^Cre^* and *Lck^Cre^*;*Rbx1*^*fl*/*fl*^ mice. (E) Expression of *Mki67* gene in clusters 1, 3, and 11 in γδ T cells of the thymus from *Lck^Cre^* and *Lck^Cre^*;*Rbx1*^*fl*/*fl*^ mice. (F) Top 10 KEGG pathways of clusters 1, 3, and 11 in γδ T cells of the thymus from *Lck^Cre^* and *Lck^Cre^*;*Rbx1*^*fl*/*fl*^ mice. (G) Cell cycle analysis of cluster 1, 3, and 11 cells in the thymus from *Lck^Cre^* and *Lck^Cre^*;*Rbx1*^*fl*/*fl*^ mice. (H) Ratios of *Ifng*^+^ and *Il17a*^+^ cells among γδ T cells in the thymus from *Lck^Cre^* and *Lck^Cre^*;*Rbx1*^*fl*/*fl*^ mice.

Finally, we investigated the effect of *Rbx1* deletion on γδ T cell subtypes in the thymus. To this end, we sorted γδ T cells (CD45^+^TCRγ/δ^+^TCRβ^−^) from the thymus of *Lck^Cre^* control and *Lck^Cre^*;*Rbx1*^*fl*/*fl*^ mice by FACS, followed by single-cell RNA sequencing. Our single-cell RNA sequencing data, displayed in UMAP, revealed that γδ T cells in the thymus comprised 29 subpopulations (Fig. 7A). Their maker genes were displayed in Table S3, and we selected the top 1 or top 5 definitive markers for each subpopulation to define their characteristics, as shown in Fig. 7B and Fig. S9A.

A previous study compared the single-cell transcriptome data of γδ T cells from thymus, spleen, and lymphocyte nodes in mice, and showed that the mature γδ T cells can be further divided into γδ T1 cells and γδ T17 cells, which have high expression of *Ifng* and *Il17a*, respectively, and identified an immature subpopulation of γδ T cells specially in the thymus that expresses *Gzma*, and does not express either *Ifng* nor *Il17a* [50]. In this study, we found that *Gzma* was highly expressed selectively in clusters 1, 3, 11, and 14 (Fig. 7C and Fig. S9), while *Ifng* was also highly expressed in cluster 14 (Fig. 7C), suggesting that cluster 14 did not belong to the immature γδ T cells. In clusters 1, 3, and 11, *Gzma* was highly expressed, whereas *Ifng* or *Il17a* was barely expressed (Fig. 7C), suggesting that they belonged to immature *Gzma*^+^ γδ T cells. The ratio of *Gzma*^+^ immature γδ T cells to total γδ T cells increased upon *Rbx1* deletion (17.17% in *Lck^Cre^* mice versus and 23.89% in *Lck^Cre^*;*Rbx1*^*fl*/*fl*^ mice), suggesting that *Rbx1-*deficient γδ T cells in the thymus are more immature.

Next, we examined alterations in the *Gzma*^+^ immature γδ T cell subpopulation. Notably, within the *Rbx1*-deficient *Gzma*^+^ immature γδ T cell population, ratios of clusters 1 and 3 were elevated, while cluster 11 was decreased (Fig. 7D). Among them, marker genes and enriched pathways were both similar between clusters 1 and 3 but distinct in cluster 11 (Fig. S10A and Fig. 7E), which presented an intuitive cognition that clusters 1 and 3 shared similar transcriptome characteristics, while cluster 11 was distinct from clusters 1 and 3. We further conducted an unbiased correlation analysis on clusters 1, 3, and 11; the Pearson correlation coefficient between clusters 1 and 3 is 0.973, which is obviously higher than those between clusters 11 and 1, and clusters 11 and 3 (0.922 and 0.915, respectively), indicating a distinctive characteristic between cluster 11 and clusters 1 and 3 (Fig. S11). Mechanistically, cell cycle, oxidative phosphorylation, DNA replication, and spliceosome pathways were enriched in cluster 11, while Wnt and TCR pathways were enriched in clusters 1 and 3 (Fig. 7E). Since *Rbx1* deletion resulted in a decrease of cluster 11 and elevation of clusters 1 and 3 (Fig. 7D), it is reasonable to speculate that Rbx1 might up-regulate the pathways including cell cycle, oxidative phosphorylation, DNA replication, and spliceosome, but down-regulate Wnt and TCR pathways to modulate the immature *Gzma*^+^ γδ T cells in the thymus.

Cluster 11 had high expressed *Mki67* (Table S3, Fig. S10, and Fig. 7F), a marker of proliferation. KEGG pathway analysis revealed a robust increase of pathways related to cell cycle, oxidative phosphorylation, DNA replication, and spliceosomes in cluster 11, all of these pathways are tightly associated with cell proliferation and division (Fig. 7E). Cell cycle analysis also revealed a marked increase in the S and G2/M score (Fig. 7G), and a significant increase in cell cycle-related genes in cluster 11, as compared to clusters 1 and 3 (Fig. S12). Thus, cluster 11 appears to be a proliferative subpopulation of *Gzma*^+^ immature γδ T cells. For the first time, we showed that *Gzma*^+^ immature γδ T cells contain a proliferative subpopulation, which is reduced upon *Rbx1* deletion.

We further examined the effect of Rbx1 on mature subpopulations in γδ T cells in the thymus. Clusters 2, 14, 21, 23, and 28 had higher expressed levels of *Ifng*, indicating that they are γδ T1 cells, whereas clusters 4, 7, 20, and 27 expressed higher levels of *Il17a*, belonging to γδ T17 cells (Fig. 7C). In the thymus of *Lck^Cre^* control mice, γδ T1 and γδ T17 comprised 8.23% and 17.96%, respectively, of total γδ T cells, whereas the percentage increased to 13.72% or decreased to 11.75%, respectively, in *Lck^Cre^*;*Rbx1*^*fl*/*fl*^ mice (Fig. 7H). Consistently, the ratio of γδ T1 to γδ T17 cells in the thymus was also changed markedly upon *Rbx1* deletion (0.46 in *Lck^Cre^* control mice versus 1.17 in *Lck^Cre^*;*Rbx1*^*fl*/*fl*^ mice), suggesting that *Rbx1* may determine the fate of T1 versus T17 subtype of γδ T cells in the thymus. Since γδ T1 and γδ T17 cells are functionally distinct, our findings suggest that Rbx1 controls the function of γδ T cells by determining their fate.

## Discussion

In this study, we systematically examined, using 4 conditional knockout mouse models, the function of the neddylation–CRL system in the thymus development. We identified Rbx1, but not neddylation E2, Ube2f or Ube2m, or Sag/Rbx2, as a pivotal regulator. The thymus in *Lck^Cre^*;*Rbx1*^*fl*/*fl*^ mice showed obvious atrophy (Fig. 3A and B and Fig. S1E and F), an important event characteristic of an aging of immune system, which is itself associated with reduced immunity and multiple immune-related diseases [51]. From a classical biochemical viewpoint, neddylation E2 Ube2m is essential for Rbx1 to neddylate cullins 1 to 4 [11]. The fact that, unlike *Rbx1* deletion, *Ube2m* deletion has no obvious phenotypic changes indicates that Rbx1 is independent of Ube2m, and Rbx1-associated CRL1 to CRL4 E3, but not Ube2m-associated neddylation E3, is required for proper thymus development. This is in contrast to our previous study in Treg cells, in which Rbx1 function is partially dependent on Ube2m [19–21]. Thus, it appears that the dependence of neddylation E3/Rbx1 on neddylation E2/Ube2m is determined by cellular and tissue context. Finally, the lack of phenotypical changes upon deletion of *Ube2f* or *Sag* in Treg cells [19] and the thymus (this study) indicated that the Ube2f–Sag axis plays no physiological role in the maintenance of Treg cells and in the development of the thymus.

The main function of RBX1-CRLs E3 is to promote ubiquitylation and subsequence proteasome degradation of substrate proteins [9]. To this end, we have focused on Rbx1 substrate Bim [52], a pro-apoptotic protein, for its possible involvement. Indeed, we found that thymus *Rbx1* deletion caused Bim accumulation (Fig. 4A and B). However, accumulated Bim plays a minor, if any, role in defective thymus development caused by *Rbx1* deletion, since simultaneous deletion of *Bim* did not rescue the severe phenotypes (Fig. 4 and Fig. S2). Thus, the function of Rbx1 in thymus development is not simply to ensure the survival or prevent apoptosis. On the other hand, it is not a surprise that a single substrate failed to rescue the defective phenotypes, given that Rbx1 has many substrates that control a variety of biological processes [9].

While *Rbx1* deficiency delayed the thymus development in general, more detailed cellular study by FACS analysis showed that *Rbx1* deficiency actually causes accumulation of DN cells (Fig. 3). The single-cell RNA sequencing analysis of DN3 and DN4 cells revealed that Rbx1 regulates differentiation of these cell populations. Upon *Rbx1* deletion, ratios of clusters 3, 4, and 9 in DN3 were elevated, and ratios of clusters 11, 12, and 13 in DN4 were decreased, while other clusters were little altered in their portions (Fig. 5C). These results uncovered the detailed mechanism of Rbx1 on thymus development. Very few studies were reported using single-cell RNA sequencing for thymus. Examples include single-cell transcriptomic atlases of the thymus from human [53,54] or mice [55] in embryonic stages. Our study focuses on the DN3&4 cells in the thymus from adult mice, and the data generated will broaden our current understanding of thymus development at the molecular levels [56].

How the fate of αβ/γδ T cells in the thymus is determined is a pivotal issue in the field of immunology. While the TCR pathway is known to play an important role [6], few recent studies showed that the signaling pathways regulated by Sox13 [57], Plzf [58], Id3 [59], mTOR [47], and RNA m^6^A methylation [60] are also involved. Here, we identified Rbx1 as another important regulator of γδ T cell generation in the thymus, adding an E3 ubiquitin ligase to the list. Does Rbx1 has any connections to other known regulators of γδ T cells? Notably, Plzf was reported to associate with cullin 3, thus likely a substrate of Rbx1-CRL3 [61]. The demethylase enzymes of RNA m^6^A ALKBH5 and CRL1 were connected by circular RNA circAFF2 in colorectal cancer [62], indicating a direct connection with Rbx1, since RBX1 is a RING component of CRL1. In the transcriptome of γδ T cell progenitor DN3a cells, we found that many signaling pathways, such as phosphatidylinositol 3-kinase–AKT pathway and metabolic pathways, such as glutathione metabolism were altered upon *Rbx1* deletion, suggesting that Rbx1 precisely regulates these pathways to ensure proper development of γδ T cells. It is well known that immune responses are subjected to metabolic regulation [63,64]. Our work suggested an essential role of Rbx1 in regulation of various signaling and metabolism pathways during the development of γδ T cells.

Our single-cell RNA sequencing analysis of γδ T cells in the thymus revealed that the immature *Gzma*^+^ γδ T cells [50] can be divided into 3 subpopulations, including a proliferative subpopulation (Fig. 7). In *Rbx1*-deficient mice, the percentage of immature *Gzma*^+^ γδ T cells was elevated, while that of the proliferative subpopulation decreased. The data presented herein show, for the first time, the existence of the proliferative subpopulation in immature *Gzma*^+^ γδ T cells, which is subjected to Rbx1 regulation. We further found that Rbx1 also regulates mature γδ T cells in the thymus, since *Rbx1* deletion alters the ratios of γδ T1 cells (*Ifng*^hi^) and γδ T17 cells (*Il17a*^hi^) (Fig. 7H). Thus, it appears that Rbx1 regulates T1/T17 differentiation of γδ T cells and likely the function of γδ T cells in vivo.

**Fig. 8.**
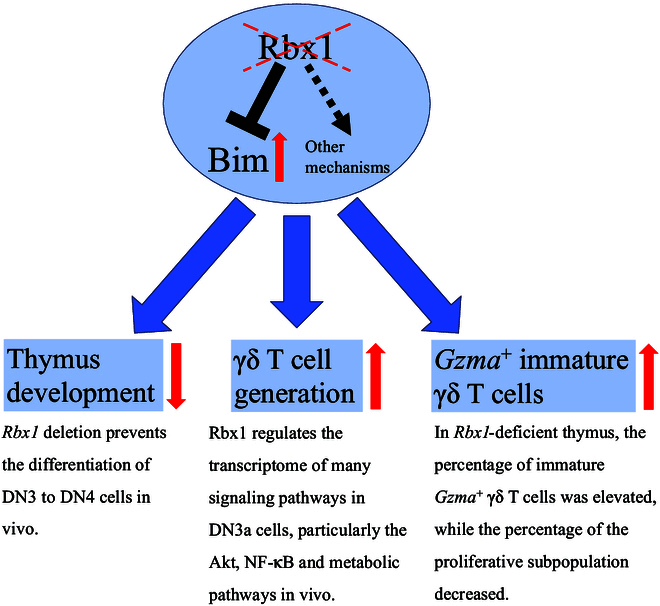
Working model for this study. We found in this study that Rbx1 is essential and required for thymus development and fate determination of αβ-γδ T cells. Bim, a substrate of Rbx1, plays a minor role in the process, while the major role must be played via other yet-to-be identified mechanisms/multiple substrates, waiting to be elucidated via future investigation.

In summary, we demonstrate here that Rbx1, but not Ube2f, Ube2m, or Sag, plays a pivotal role in regulating the development of the thymus and fate differentiation of γδ T cells as well as its subpopulation (Fig. 8), which would eventually affect the function of peripheral γδ T cells and possible associated diseases, an interesting subject for future investigation.

## Materials and Methods

### Mice

The *Lck^Cre^* mice were obtained from the Jackson Laboratory (no. 003802). The *Ube2m*-*flox* [19], *Ube2f*-*flox* [19], *Rbx1*-*flox* [19], and *Sag*-*flox* [65] mice were constructed previously. The *Bim-flox* mice were purchased from GemPharmatech Co. Ltd., Nanjing, China (no. T007185) as described [33].

Mice were fed in specific pathogen-free conditions. All animal experiments were approved by the Animal Ethics Committee of the Second Affiliated Hospital, Zhejiang University School of Medicine; animal care was provided in accordance with the principles and procedures by the regulatory standards at Zhejiang University Laboratory Animal Center.

### Flow cytometry

The thymus from indicated mice were homogenized into single cells and were further washed in phosphate-buffered saline (PBS) containing 2% (w/v) fetal bovine serum (FBS). Cells were stained with indicated antibodies for analysis of surface proteins and were fixed and permeabilized with Pharmingen Transcription Factor Buffer Set (BD Pharmingen, 562574) for intracellular proteins. Flow cytometry was performed on CytoFLEX LX (Beckman).

Antibodies used were listed as follows: anti-CD45 (S18009, BioLegend), anti-CD4 (RM4-5, BD Pharmingen), anti-CD8α (53-6.7, BioLegend), anti-CD44 (IM7, BioLegend), anti-CD25 (PC61, BioLegend), anti-TCRβ (H57-597, BioLegend), anti-TCRγ/δ (GL3, BioLegend), anti-CD28 (37.51, BioLegend), and anti-Bim (C34C5, Cell Signaling Technology).

### Cell counting

The thymus from the indicated mice were homogenized into single-cell suspensions and resuspended in 10 ml of PBS containing 2% (w/v) FBS. Cell concentrations were determined using a Countess II FL Automated Cell Counter, with total thymic cellularity calculated by multiplying the suspension volume by the corresponding cell concentration for each sample. Subsequently, parts of these cell suspensions were subjected to FACS analysis to quantify the proportions of distinct cellular subpopulations among the total cell population. Absolute cell numbers of each subpopulation were then derived by multiplying the total thymocyte count by the percentage representation of each specific population.

### Cell sorting

The thymus from indicated mice was ground into single cells in RPMI 1640 medium containing 2% (w/v) FBS. Then, cells were stained with different antibodies, followed by FACS, performed on SONY Cell Sorter (SH800S). All the harvested cells were detected with purities >99%.

### RNA extraction and qPCR

The CD45^+^CD4^−^CD8^−^CD44^−^ DN3&4 cells were sorted from the thymus of indicated mice and were subjected to RNA isolation using Total RNA Kit I (Omega, R6834). Then, complementary DNA (cDNA) was reversely transcribed from RNA by using the PrimeScript RT reagent Kit (Takara, RR037A), and followed by quantitative polymerase chain reaction (qPCR) using SYBR Premix Ex Taq (TaKaRa, RR420B) on an Applied Biosystems ViiA real-time PCR system (Applied Biosystems, Waltham, MA).

The sequences of the primer pairs used in qPCR are as follows: *Sag*: 5′-TGGAGGACGGCGAGGAAC-3′ and 5′-CCCCAGACCACAACACAGTC-3′, *Ube2f*: 5′-GACTGTAAGCCCAGATGAG-3′ and 5′-CCTTTAATGTTCTAGTGGG-3′, *Ube2m*: 5′-CTGTCCTGATGAAGGCTTC-3′, and 5′-GTTCGGCTCCAAGAAGAG-3′, *Rbx1*: 5′-CTTTGTATCGAATGTCAGGC-3′ and 5′-GTCACTAGACGAGTAACAG-3′; *Gapdh*: 5′-GCCGCCTGGAGAAACCTGCC-3′ and 5′-GGTGGAAGAGTGGGAGTTGC-3′ as described previously [19].

### Western blot

The antibodies used in Western blot were as follows: anti-Sag, Proteintech, 11905-1-AP; anti-Ube2f, Proteintech, 17056-1-AP; anti-Ube2m, Santa Cruz Biotechnology, sc-390064; and anti-β-actin, HuaBio, EM21002.

### Bulk RNA sequencing

The CD4^−^CD8^−^CD44^−^CD25^+^CD28^−^ DN3a cells were sorted from the thymus of *Lck^Cre^* or *Lck^Cre^*;*Rbx1*^*fl*/*fl*^ mice, respectively. Leveraging the structural feature that most mRNAs in eukaryotic organisms possess a polyA tail, the experiment employs an Oligo(dT)VN primer as the reverse transcription primer to directly carry out cDNA synthesis in a cell lysate containing RNA. Additionally, utilizing the template-switching activity of the Sc Reverse Transcriptase enzyme, a linker sequence is added to the 3’ end of the cDNA. Subsequent PCR amplification is conducted through this linker sequence, resulting in the acquisition of full-length cDNA amplification products. These products are then subjected to quality control and fragment sorting through electrophoresis. Subsequently, the DNA is disrupted using a modified high-activity Tn5 transposase, and cDNA fragments around 200 to 300 base pairs (bp) are selected for PCR amplification and subsequent purification of the PCR product, ultimately yielding a library. After passing the library inspection, the library preparations were sequenced on a NovaSeq 6000 platform and 150-bp stand-specific paired-end reads were generated.

### Single-cell RNA sequencing

The CD45^+^CD4^−^CD8^−^CD44^−^ DN3&4 cells or CD45^+^TCRγ/δ^+^ TCRβ^−^ γδ T cells were sorted from the thymus of *Lck^Cre^* or *Lck^Cre^*;*Rbx1*^*fl*/*fl*^ mice, respectively. The single-cell suspension was loaded into the microfluidic devices. Subsequently, the single-cell TCR sequencing libraries were constructed according to the protocol of GEXSCOPE Single Cell Immuno-TCR Kit (Singleron Biotechnologies). In brief, the magnetic beads with molecular labels captured the poly(A) tail and TCR region on the mRNA to label the cells and mRNA after the cells are lysed. Afterward, the magnetic beads in the chip were collected, and then mRNAs captured by the magnetic beads were reverse transcribed into cDNA and amplified. Sequencing libraries suitable for NovaSeq sequencing platform were constructed after partial cDNA fragments and splicing. The remaining cDNA was enriched for the immune receptor (TCR), and the enriched products were amplified by PCR to construct a sequencing library suitable for the NovaSeq sequencing platform. Finally, each library was sequenced on NovaSeq 6000 with 150-bp paired-end reads.

### Statistical analysis

Statistical analyses were performed by GraphPad software. Statistical comparisons were analyzed using the Student’s *t* test or one-way analysis of variance (ANOVA) with data collected from independent experiments. Significant differences were considered as *P* < 0.05.

## Data Availability

The bulk RNA sequencing and single-cell RNA sequencing data generated in this study have been deposited into CNGB Sequence Archive (CNSA) [66] of China National GeneBank DataBase (CNGBdb) [67] with accession numbers CNP0007155, CNP0007156, and CNP0007184, respectively.
