# Supplementary material for: E3 Ligase Rbx1 Orchestrates Thymus Development and Fate Determination of αβ-γδ T Cells
Source: Research (Wash D C). 2025 Jul 10;8:0774. doi: 10.34133/research.0774 (PMC12241798; doi:10.34133/research.0774)
Supplement: Supplementary 1 — Figs. S1 to S12 Tables S1 to S4 [file research.0774.f1.zip › Supplementary figure.pdf]

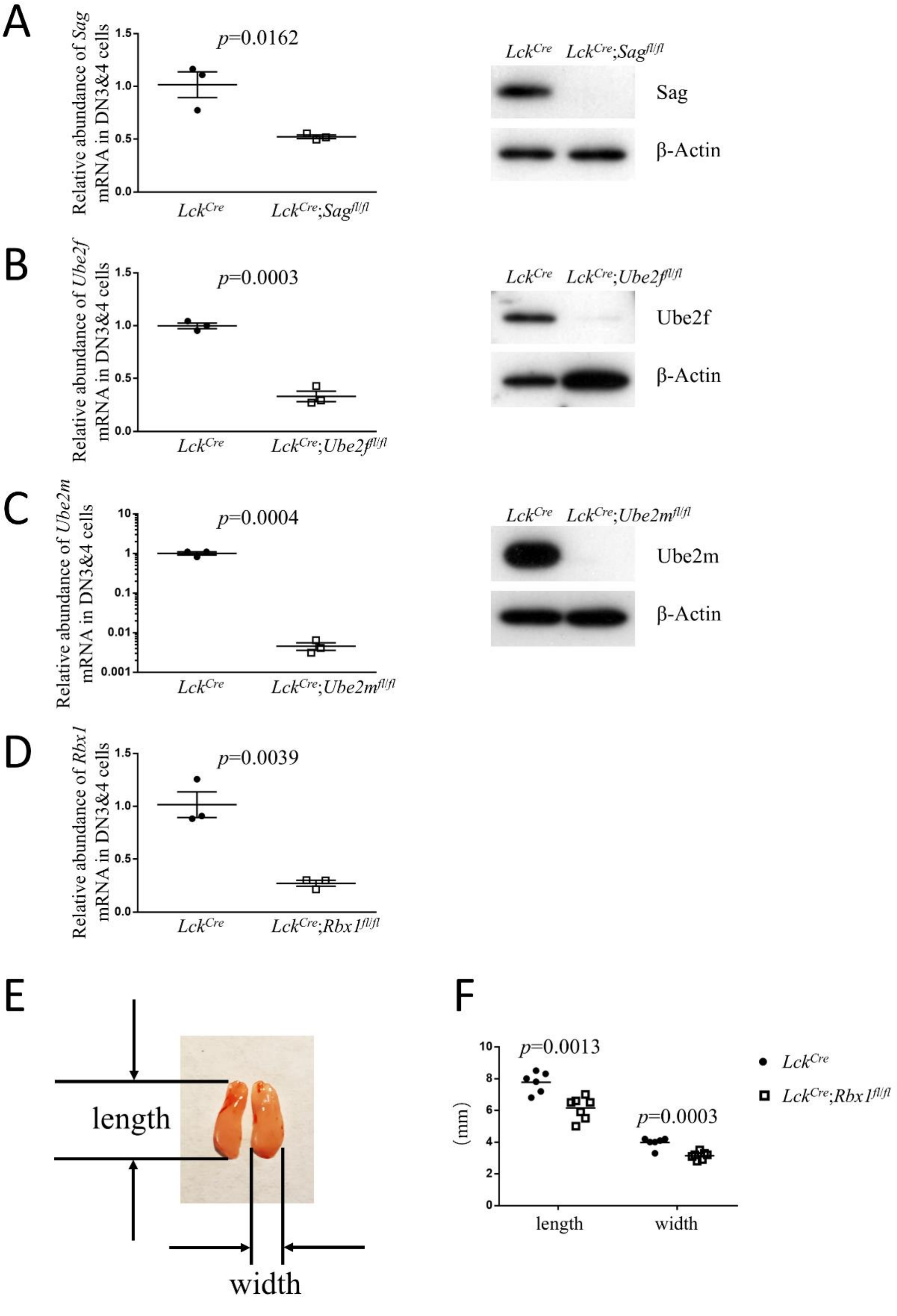
A
Relative abundance of Sag mRNA in DN3&4 cells
p=0.0162
Lck^Cre
Lck^Cre;Sag^fl/fl
Sag
β-Actin
B
Relative abundance of Ube2f mRNA in DN3&4 cells
p=0.0003
Lck^Cre
Lck^Cre;Ube2f^fl/fl
Ube2f
β-Actin
C
Relative abundance of Ube2m mRNA in DN3&4 cells
p=0.0004
Lck^Cre
Lck^Cre;Ube2m^fl/fl
Ube2m
β-Actin
D
Relative abundance of Rbx1 mRNA in DN3&4 cells
p=0.0039
Lck^Cre
Lck^Cre;Rbx1^fl/fl
E
length
width
F
(mm)
p=0.0013
p=0.0003
length
width
Lck^Cre
Lck^Cre;Rbx1^fl/fl

Supplementary Fig. 1.   knockout efficacy and the alteration of thymus size

A. q-RT-PCR (n=3) and western blotting analyses to detect the levels of Sag mRNA and proteins in DN3&4 cells from thymus of *Lck*^{Cre} and *Lck*^{Cre};*Sag*$^{fl/fl}$ mice at 8 weeks old.

B. q-RT-PCR (n=3) and western blotting analysis to detect the levels of Ube2f mRNA and proteins in DN3&4 cells from thymus of *Lck*^{Cre} and *Lck*^{Cre};*Ube2f*$^{fl/fl}$ mice at 8 weeks old.

C. q-RT-PCR (n=3) and western blotting analysis to detect the levels of Ube2m in DN3&4 cells from thymus of *Lck*^{Cre} and *Lck*^{Cre};*Ube2m*$^{fl/fl}$ mice at 8 weeks old.

D. q-RT-PCR detection of *Rbx1* mRNA in DN3&4 cells from thymus of *Lck*^{Cre} and *Lck*^{Cre};*Rbx1*$^{fl/fl}$ mice at 8 weeks old (n=3).

E. Diagram of the length and width of the thymus. To evaluate the size of the thymus, both the length and width of thymus were measured as indicated.

F. Statistical analysis of the length and width of thymus from *Lck*^{Cre} and *Lck*^{Cre};*Rbx1*$^{fl/fl}$ mice at 8 weeks old (n=6-7).

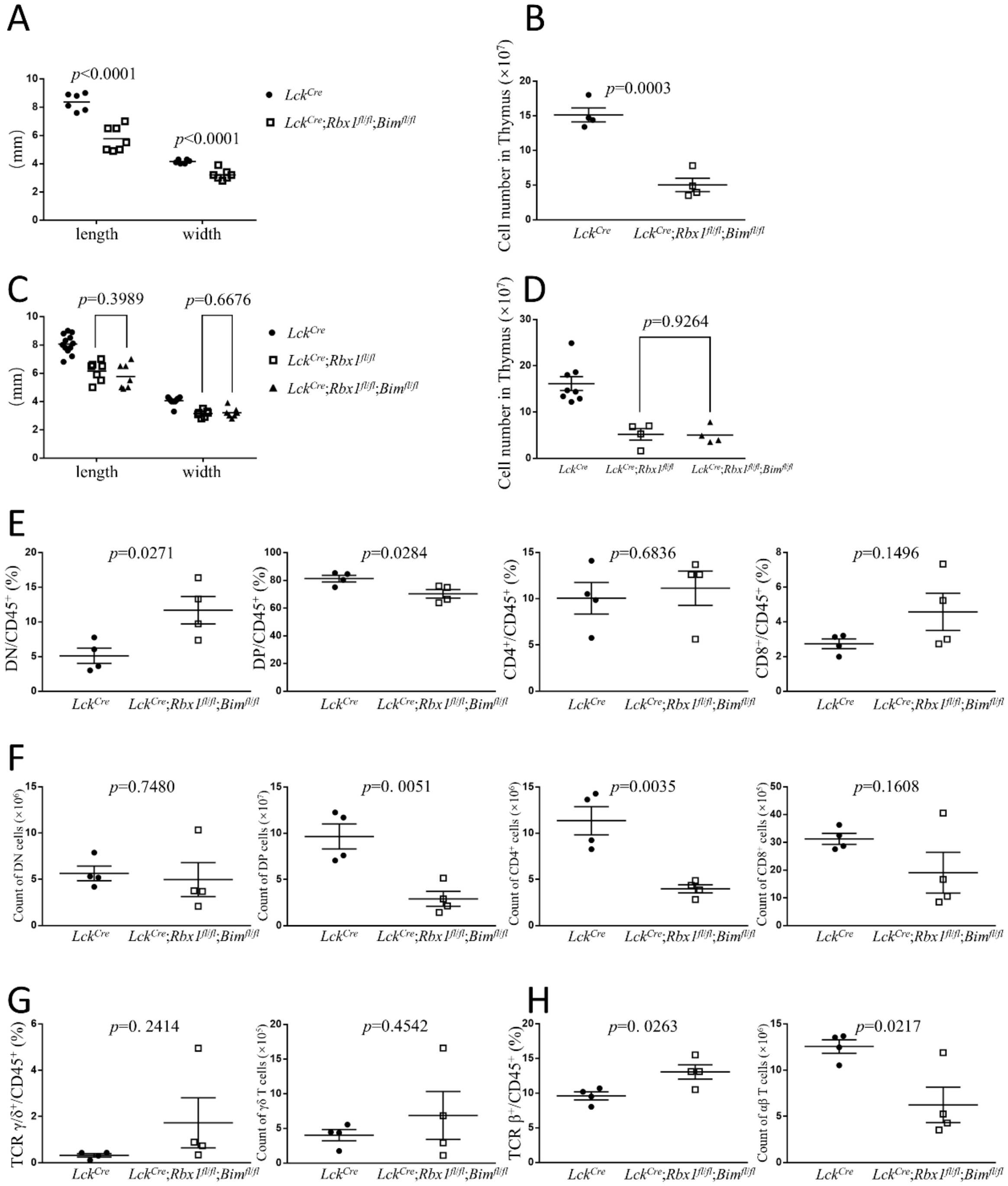

A
p<0.0001
p<0.0001
(mm)
length
width
LckCre
LckCre;Rbx1fl/fl;Bimfl/fl
B
Cell number in Thymus (×10^7)
p=0.0003
LckCre
LckCre;Rbx1fl/fl;Bimfl/fl
C
p=0.3989
p=0.6676
(mm)
length
width
LckCre
LckCre;Rbx1fl/fl
LckCre;Rbx1fl/fl;Bimfl/fl
D
Cell number in Thymus (×10^7)
p=0.9264
LckCre
LckCre;Rbx1fl/fl
LckCre;Rbx1fl/fl;Bimfl/fl
E
DN/CD45+ (%)
p=0.0271
DP/CD45+ (%)
p=0.0284
CD4+/CD45+ (%)
p=0.6836
CD8+/CD45+ (%)
p=0.1496
F
Count of DN cells (×10^6)
p=0.7480
Count of DP cells (×10^7)
p=0. 0051
Count of CD4+ cells (×10^6)
p=0.0035
Count of CD8+ cells (×10^5)
p=0.1608
G
TCR γ/δ+/CD45+ (%)
p=0. 2414
Count of γδ T cells (×10^5)
p=0.4542
H
TCR β+/CD45+ (%)
p=0. 0263
Count of αβ T cells (×10^6)
p=0.0217

Supplementary Fig. 2. Analysis of the phenotype of *Lck*^{Cre};*Rbx1*$^{fl/fl}$;*Bim*$^{fl/fl}$ mice

A. Sizes of the thymus from *Lck*^{Cre} and *Lck*^{Cre};*Rbx1*$^{fl/fl}$;*Bim*$^{fl/fl}$ mice at 8 weeks old (n=6-7).

B. Cell numbers of thymus from *Lck*^{Cre} and *Lck*^{Cre};*Rbx1*$^{fl/fl}$;*Bim*$^{fl/fl}$ mice at 8 weeks old (n=4).

C. Sizes of the thymus from *Lck*^{Cre}, *Lck*^{Cre};*Rbx1*$^{fl/fl}$ and *Lck*^{Cre};*Rbx1*$^{fl/fl}$ mice at 8 weeks old (n=12-14)

D. Cell numbers of thymus from *Lck*^{Cre}, *Lck*^{Cre};*Rbx1*$^{fl/fl}$ and *Lck*^{Cre};*Rbx1*$^{fl/fl}$;*Bim*$^{fl/fl}$ mice at 8 weeks old (n=4-8).

E. Ratios of DN, DP, SP1 and SP2 cells among $CD45^+$ cells in thymus from *Lck*^{Cre} and *Lck*^{Cre};*Rbx1*$^{fl/fl}$;*Bim*$^{fl/fl}$ mice at 8 weeks old (n=4).

F. Cell numbers of DN, DP, SP1 and SP2 cells among $CD45^+$ cells in thymus from *Lck*^{Cre} and *Lck*^{Cre};*Rbx1*$^{fl/fl}$;*Bim*$^{fl/fl}$ mice at 8 weeks old (n=4).

G. Ratios and numbers of γδ T cells in thymus from *Lck*^{Cre} and *Lck*^{Cre};*Rbx1*$^{fl/fl}$;*Bim*$^{fl/fl}$ mice at 8 weeks old (n=4).

H. Ratios and numbers of αβ T cells in thymus from *Lck*^{Cre} and *Lck*^{Cre};*Rbx1*$^{fl/fl}$;*Bim*$^{fl/fl}$ mice at 8 weeks old (n=4).

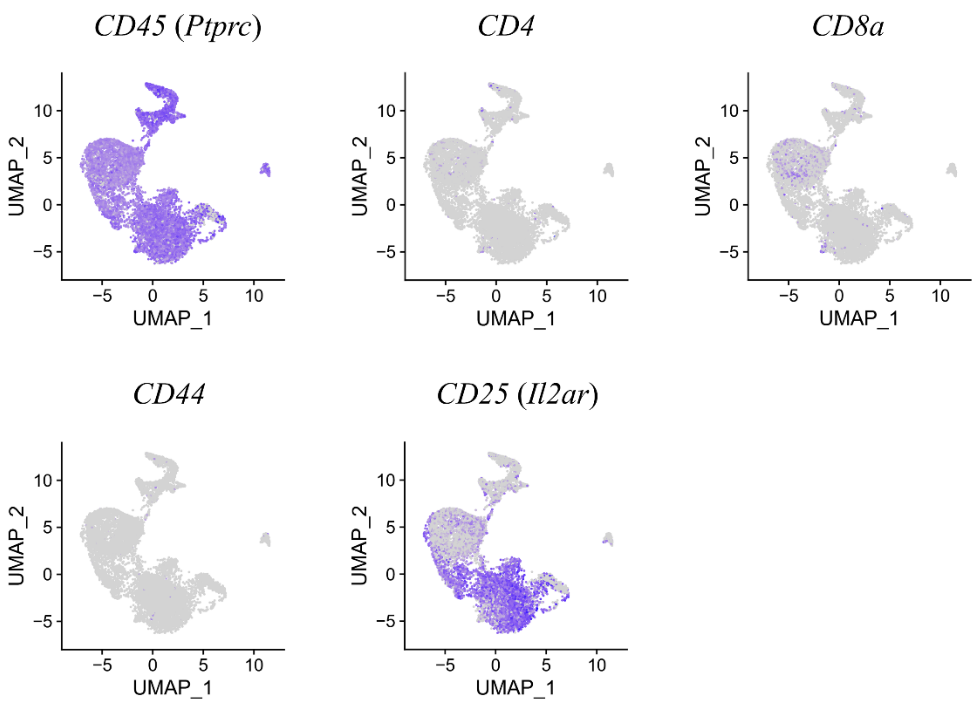

Supplementary Fig. 3. Expression of marker genes in DN3&4 cells

CD45, *CD4*, *CD8a*, *CD44* are marker genes for DN3&4 cells sorting. Here, we verified the expression of these genes in sorted DN3&4 cells by single cell RNA sequencing, and found that their expression matched with sorting condition, and further distinguishing the DN3 and DN4 cells by *Cd25* expression.

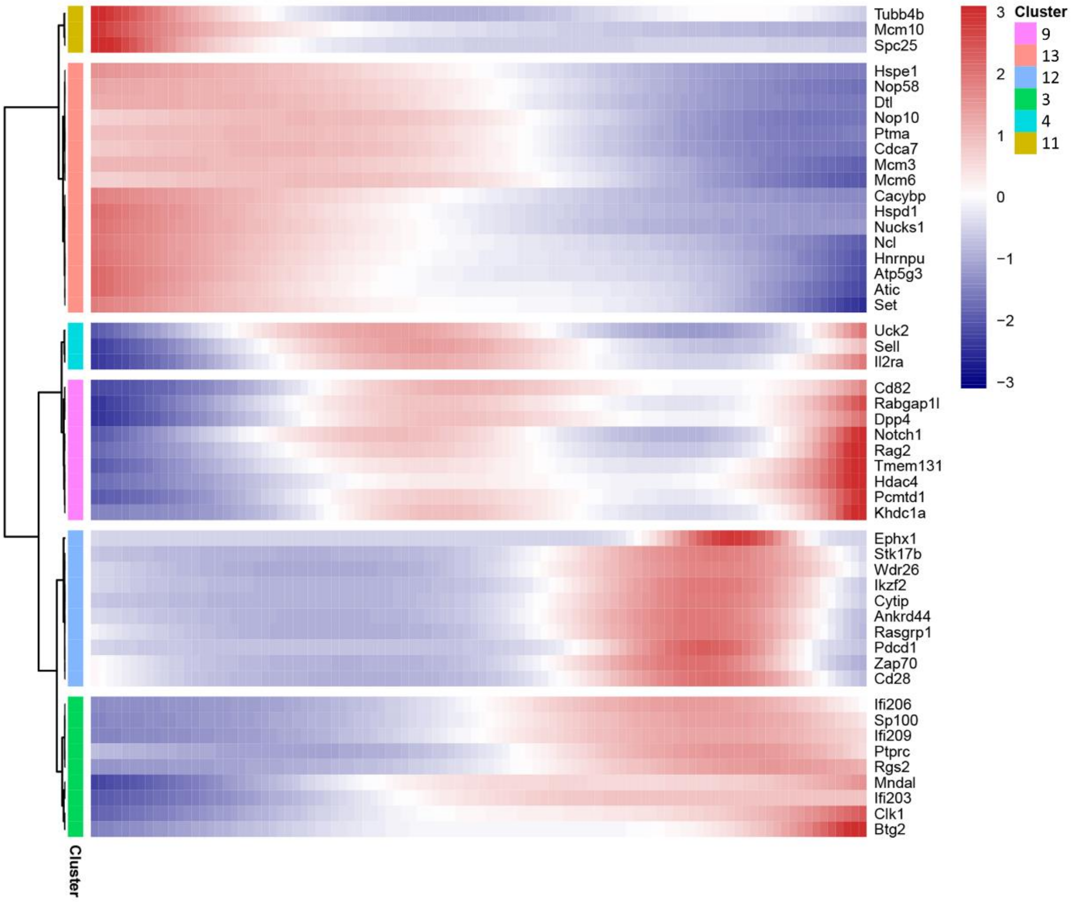

Supplementary Fig. 4. Heatmap of pseudotime analysis of clusters 3, 4, 9, 11, 12, 13 of DN3 and DN4 cells in thymus from *Lck^{Cre}* and *Lck^{Cre};Rbx1$^{fl/fl}$* mice

Shown is the alteration of the marker genes in the developmental processes in the heatmap format.

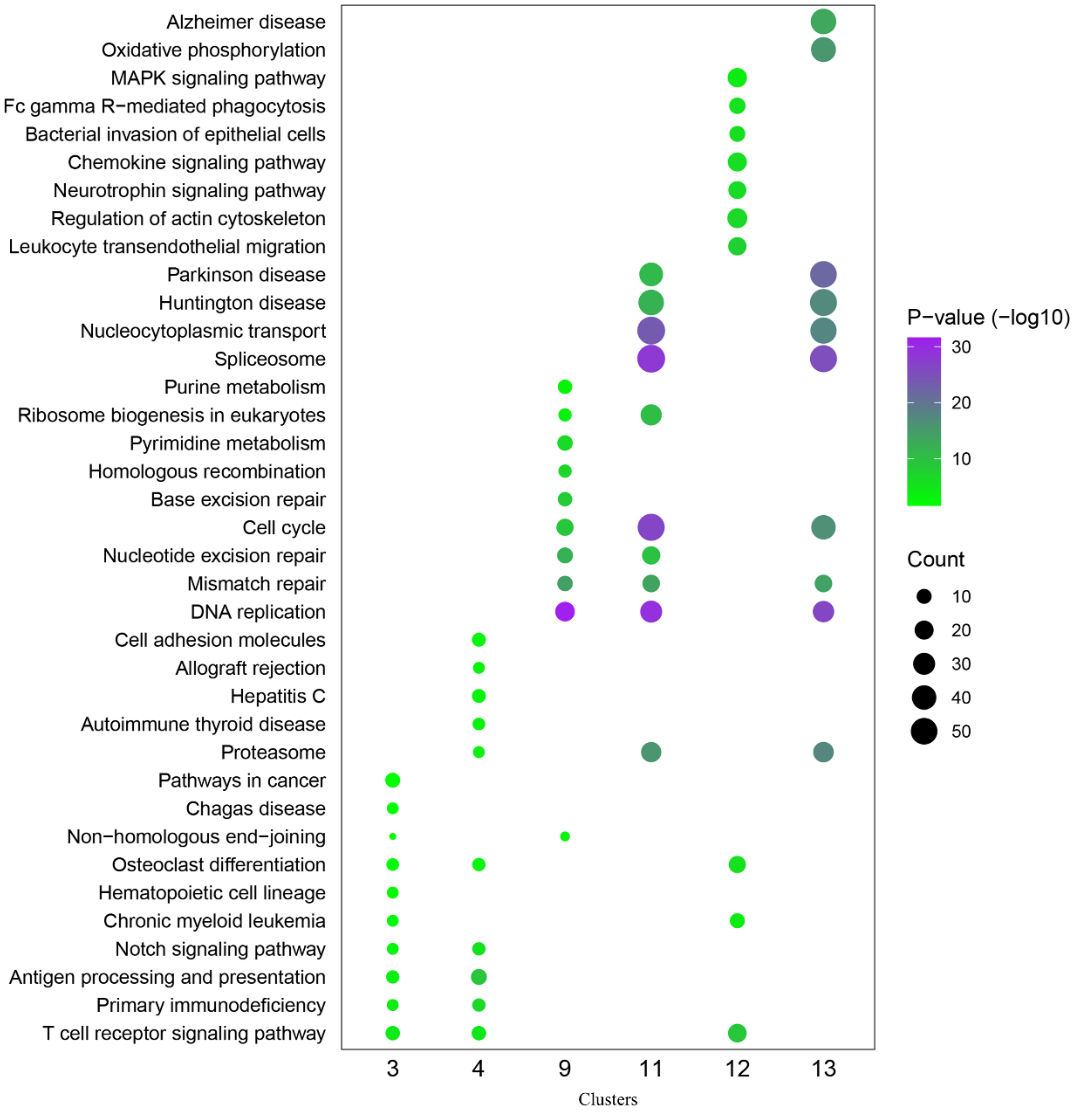

Supplementary Fig. 5. Top 10 KEGG pathways of Cluster 3, 4, 9, 11, 12, 13 of DN3 and DN4 cells in thymus from *Lck*^{Cre} and *Lck*^{Cre};*Rbx1*$^{fl/fl}$ mice

The analysis was conducted on the altered genes with |$\log_2$Fold change|>1 and p value < 0.05.

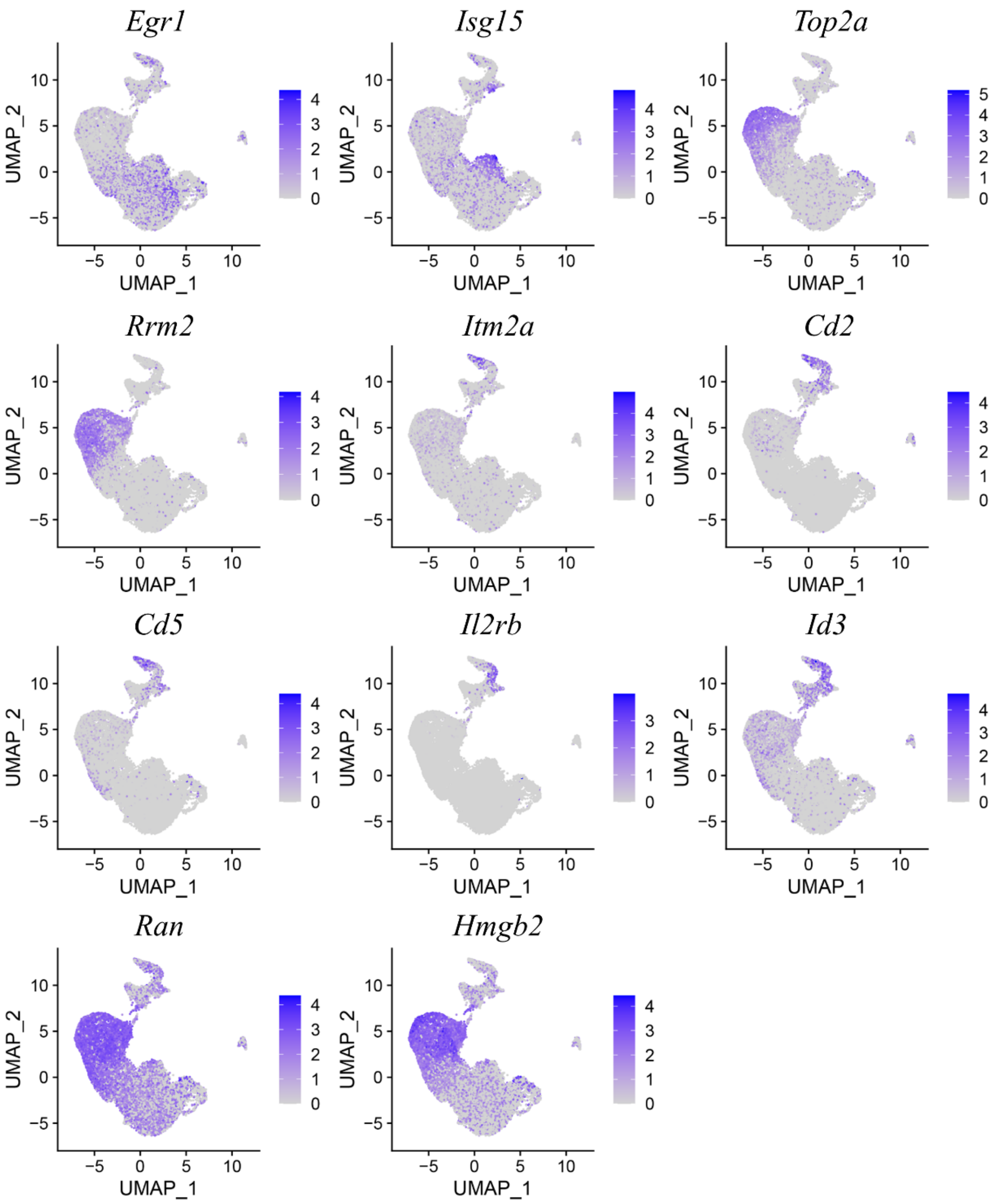

Supplementary Fig. 6.   Expression of selected genes in DN3&4 cells

Shown is the expression of selected marker genes via UMAP pattern of DN3&4 cells in thymus.

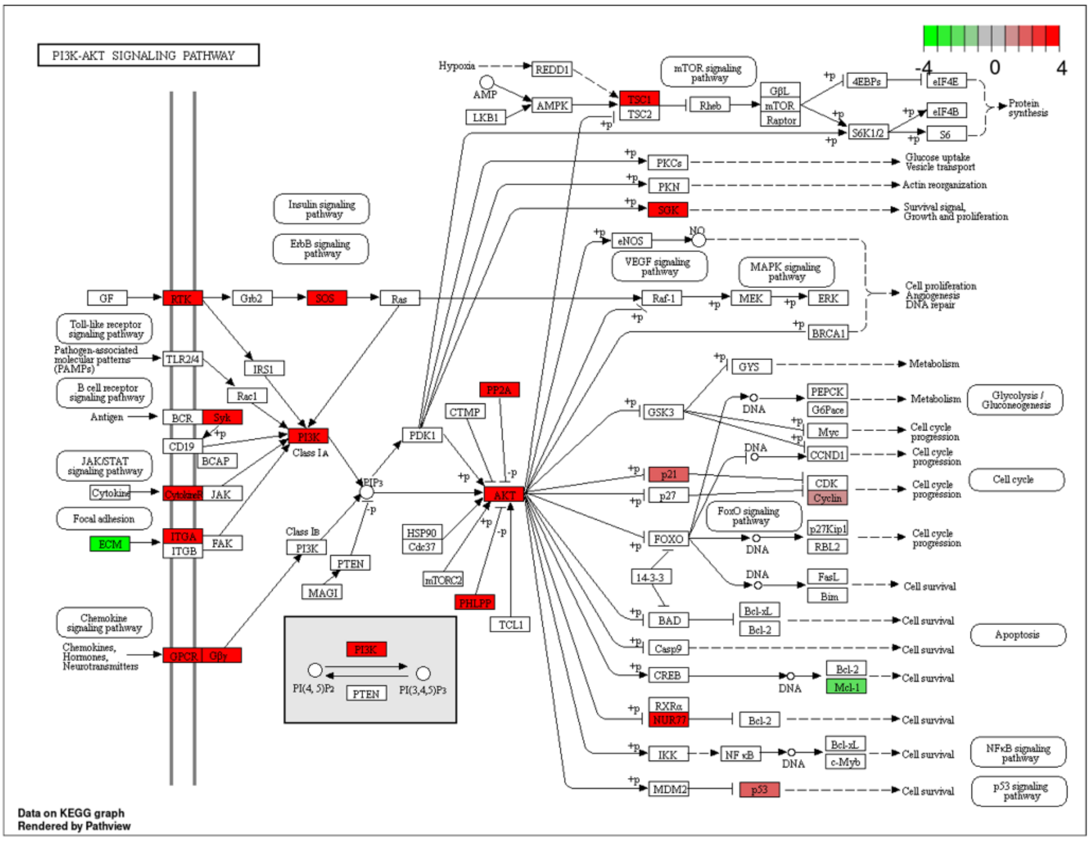
PI3K-AKT SIGNALING PATHWAY
-4
0
4
Hypoxia
REDD1
AMP
AMPK
LKB1
TSC1
TSC2
mTOR signaling pathway
Rheb
GβL
mTOR
Raptor
4EBPs
eIF4E
eIF4B
S6K1/2
S6
Protein synthesis
PKCs
Glucose uptake
Vesicle transport
PKN
Actin reorganization
SGK
Survival signal, Growth and proliferation
eNOS
NO
VEGF signaling pathway
MAPK signaling pathway
Cell proliferation
Angiogenesis
DNA repair
Insulin signaling pathway
ErbB signaling pathway
GF
RTK
Grb2
SOS
Ras
Raf-1
MEK
ERK
BRCA1
Toll-like receptor signaling pathway
Pathogen-associated molecular patterns (PAMPs)
TLR2/4
IRS1
Rac1
B cell receptor signaling pathway
Antigen
BCR
Syk
CD19
BCAP
PI3K
Class IA
PDK1
CTMP
PP2A
GYS
Metabolism
GSK3
DNA
PEPCK
G6Pase
Glycolysis / Gluconeogenesis
Myc
Cell cycle progression
CCND1
JAK/STAT signaling pathway
Cytokine
CytokineR
JAK
PIP3
AKT
p21
p27
CDK
Cyclin
Cell cycle
Focal adhesion
ECM
ITGA
ITGB
FAK
Class IB
PI3K
PTEN
MAGI
HSP90
Cdc37
mTORC2
PHLPP
TCL1
FoxO signaling pathway
FOXO
p27Kip1
RBL2
14-3-3
FasL
Bim
Cell survival
BAD
Bcl-xL
Bcl-2
Apoptosis
Chemokine signaling pathway
Chemokines, Hormones, Neurotransmitters
GPCR
Gβγ
PI(4,5)P2
PI(3,4,5)P3
Casp9
CREB
Bcl-2
Mcl-1
RXRα
NUR77
IKK
NF-κB
c-Myb
NFκB signaling pathway
MDM2
p53
p53 signaling pathway
Data on KEGG graph
Rendered by Pathview

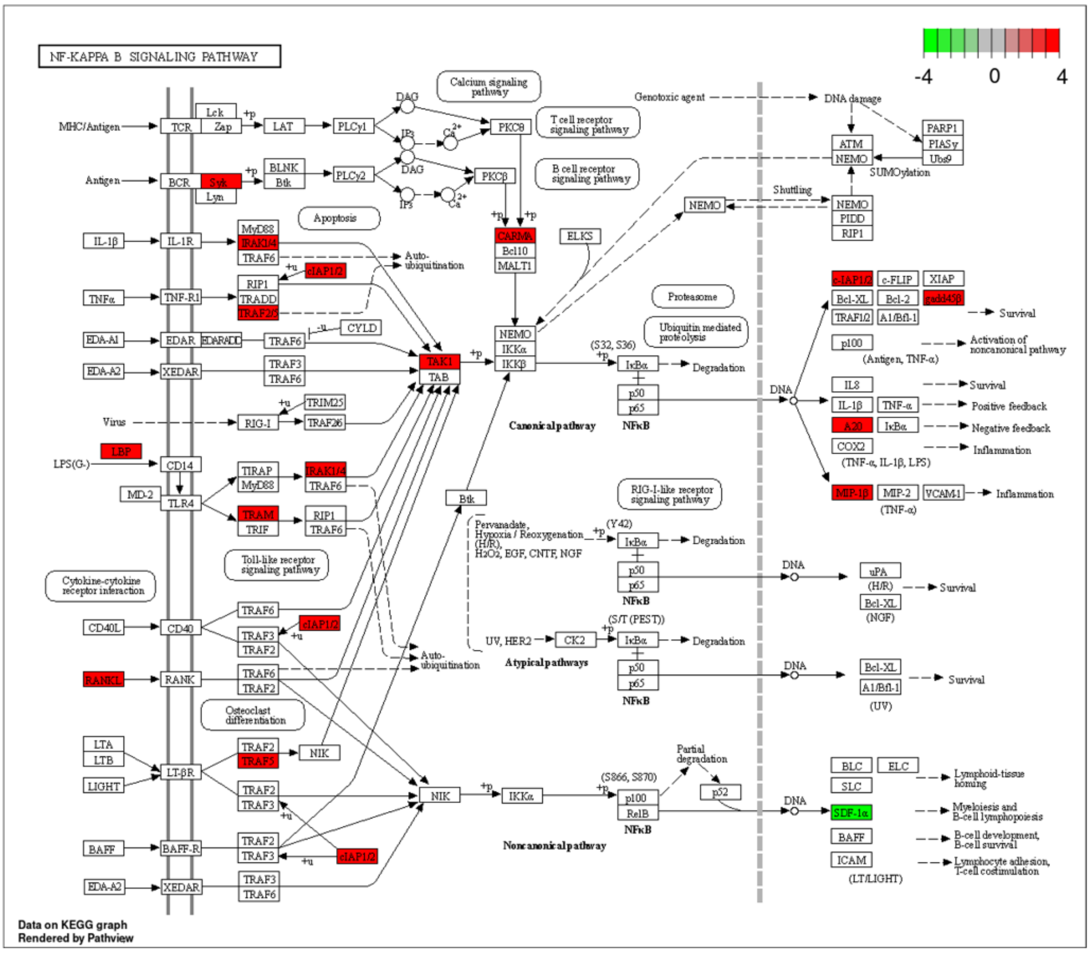
NF-KAPPA B SIGNALING PATHWAY
-4
0
4
Calcium signaling pathway
MHC/Antigen
TCR
Lck
Zap
LAT
PLCγ1
DAG
IP3
Ca2+
PKCθ
T cell receptor signaling pathway
Genotoxic agent
DNA damage
ATM
NEMO
PARP1
PIASy
Ubc9
SUMOylation
Antigen
BCR
Syk
Lyn
BLNK
Btk
PLCγ2
PKCβ
B cell receptor signaling pathway
Shuttling
NEMO
PIDD
RIP1
IL-1β
IL-1R
MyD88
IRAK1/4
TRAF6
Apoptosis
Auto-ubiquitination
CARMA
Bcl10
MALT1
ELKS
TNFα
TNF-R1
RIP1
TRADD
TRAF2/5
cIAP1/2
Proteasome
Ubiquitin mediated proteolysis
c-IAP1/2
c-FLIP
XIAP
Bcl-XL
Bcl-2
gadd45β
TRAF1/2
A1/Bfl-1
Survival
p100
(Antigen, TNF-α)
Activation of noncanonical pathway
EDA-A1
EDAR
EDARADD
TRAF6
CYLD
NEMO
IKKα
IKKβ
(S32, S36)
IκBα
Degradation
p50
p65
NFκB
EDA-A2
XEDAR
TRAF3
TRAF6
TAK1
TAB
Virus
RIG-I
TRIM25
TRAF2/6
Canonical pathway
DNA
IL8
IL-1β
TNF-α
A20
IκBα
COX2
Positive feedback
Negative feedback
Inflammation
(TNF-α, IL-1β, LPS)
LBP
LPS(G-)
CD14
MD-2
TLR4
TIRAP
MyD88
IRAK1/4
TRAF6
TRAM
TRIF
RIP1
TRAF6
Btk
RIG-I-like receptor signaling pathway
MIP-1β
MIP-2
VCAM-1
(TNF-α)
Pervanadate, Hypoxia / Reoxygenation (H/R), H2O2, EGF, CNTF, NGF
(Y42)
uPA
(H/R)
Bcl-XL
(NGF)
Toll-like receptor signaling pathway
Cytokine-cytokine receptor interaction
CD40L
CD40
TRAF6
TRAF3
TRAF2
cIAP1/2
UV, HER2
CK2
(S/T (PEST))
Atypical pathways
Bcl-XL
A1/Bfl-1
(UV)
RANKL
RANK
TRAF6
TRAF2
Osteoclast differentiation
LTA
LTB
LIGHT
LT-βR
TRAF2
TRAF5
NIK
TRAF2
TRAF3
NIK
IKKα
(S866, S870)
p100
RelB
Partial degradation
p52
Noncanonical pathway
BLC
ELC
SLC
SDF-1α
BAFF
ICAM
(LT/LIGHT)
Lymphoid-tissue homing
Myeloiesis and B-cell lymphopoiesis
B-cell development, B-cell survival
Lymphocyte adhesion, T-cell costimulation
BAFF
BAFF-R
TRAF2
TRAF3
cIAP1/2
EDA-A2
XEDAR
TRAF3
TRAF6
Data on KEGG graph
Rendered by Pathview

Supplementary Fig. 7 Akt and Nf-κb pathways in the KEGG pathway analysis of the transcriptional data in *Rbx1*-deficient DN3a cells

Rbx1 deficiency leads to alterations of numerous pathways; among them, Akt and Nf-κb pathways are known to be related with T cell regulation. Shown is the changes of Akt and Nf-κb pathway genes with cut-off criterion of ($|\log_2$Fold change$|>1$ and p value < 0.05).

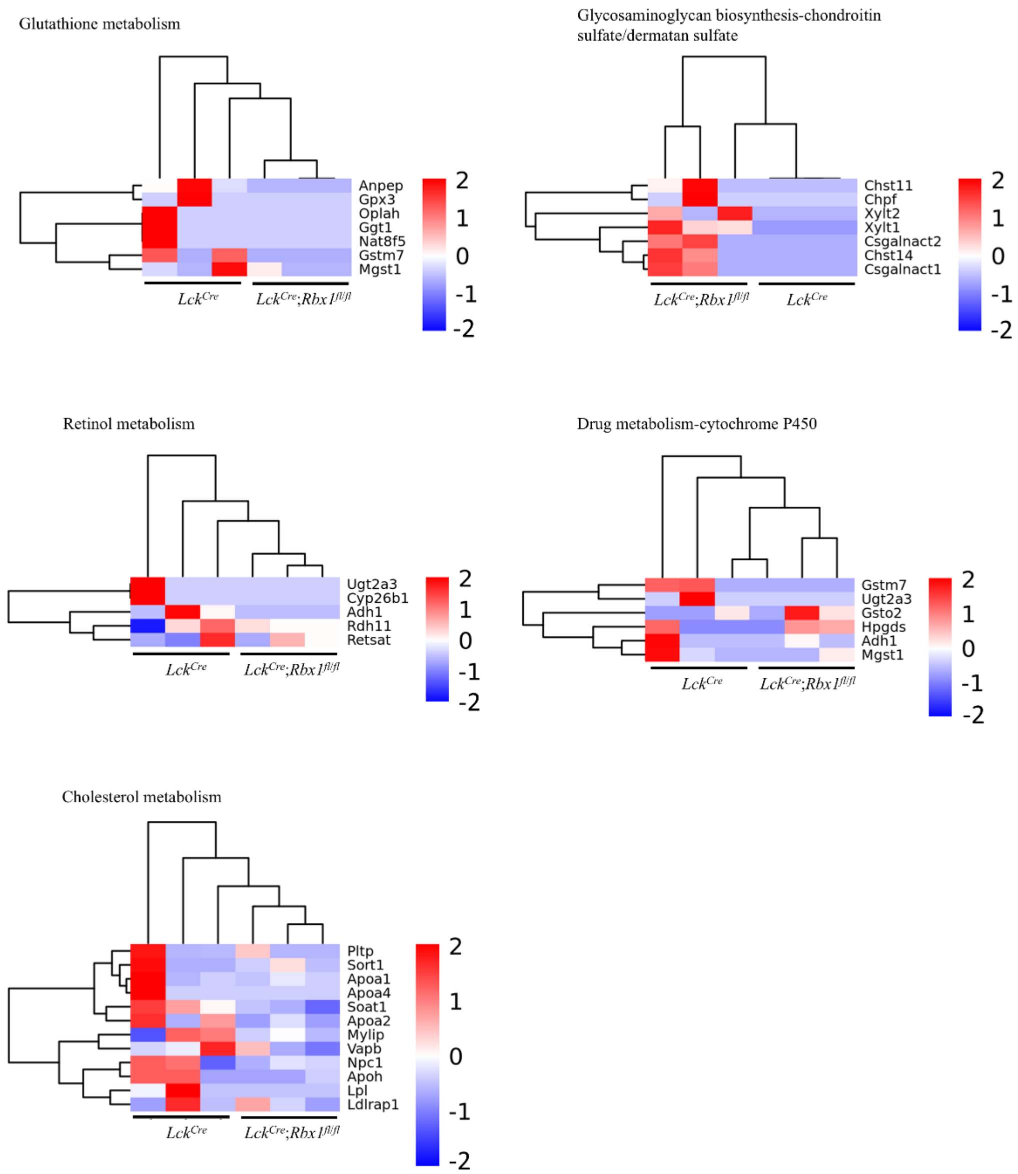

Supplementary Fig. 8. Heatmaps of the selected GSEA pathway analysis of the transcriptional data in *Rbx1*-deficient DN3a cells

For more details, see Fig. 6C.

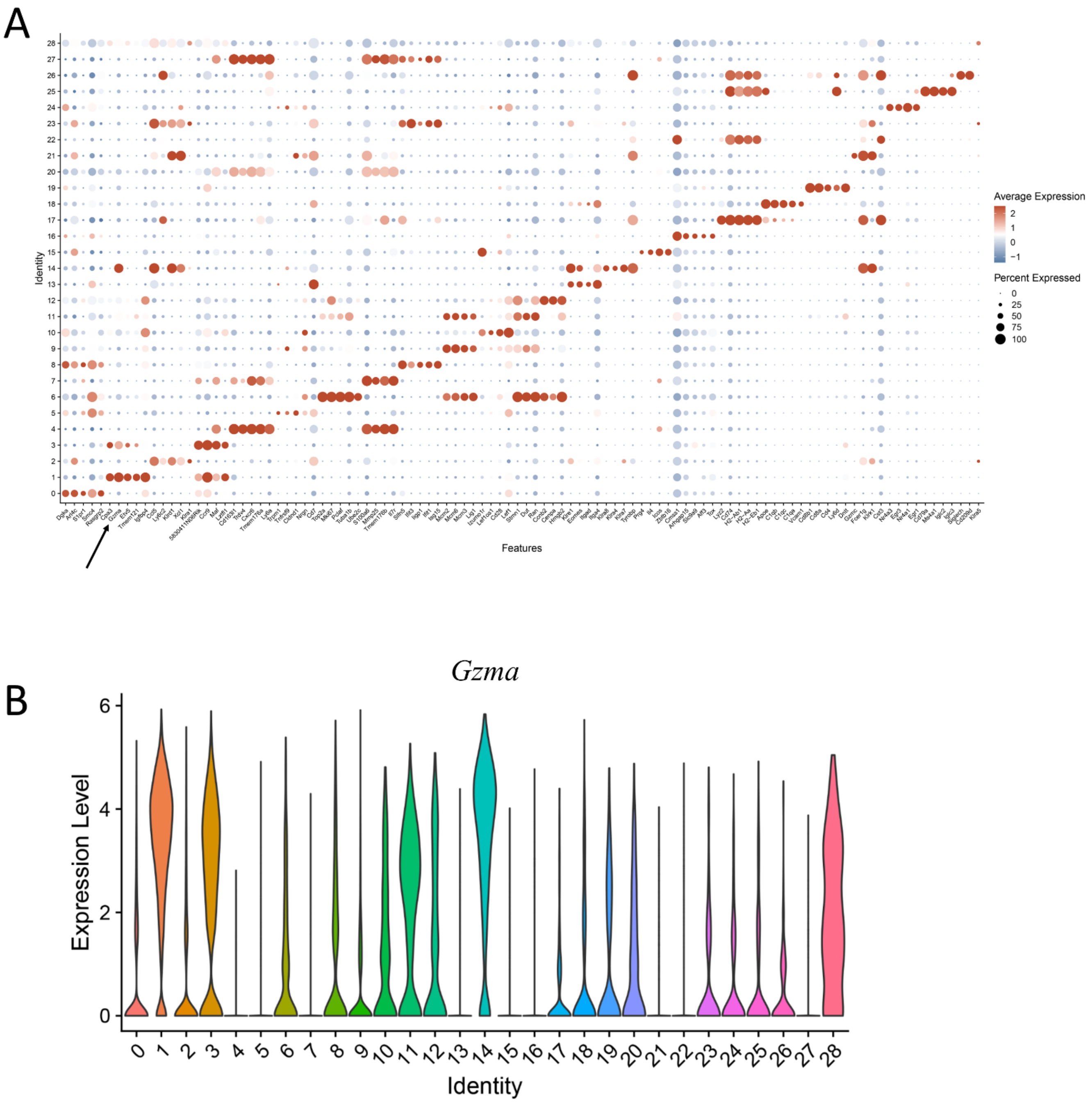

Supplementary Fig. 9. Expression of marker genes of each cluster of γδ T cells in thymus from *Lck*^{Cre} and *Lck*^{Cre};*Rbx1*$^{fl/fl}$ mice

A. Top 5 marker genes of each cluster of γδ T cells in thymus from *Lck*^{Cre} and *Lck*^{Cre};*Rbx1*$^{fl/fl}$ mice (*Gzma* was indicated by arrow).

B. Expression of *Gzma* in each cluster of γδ T cells in thymus from *Lck*^{Cre} and *Lck*^{Cre};*Rbx1*$^{fl/fl}$ mice.

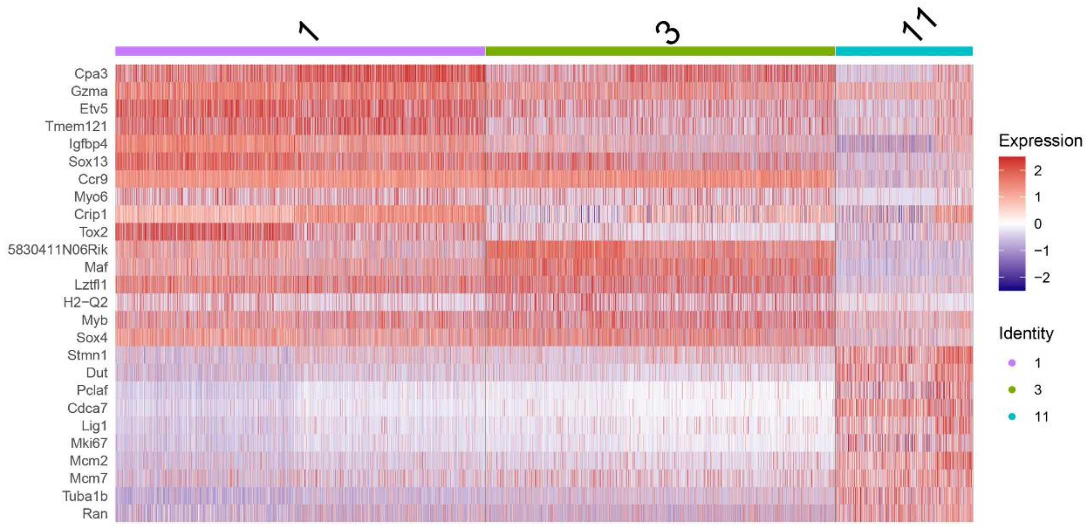

Supplementary Fig. 10. Top 10 marker genes of Cluster 1, 3, 11 of γδ T cells in thymus from *Lck*^{Cre} and *Lck*^{Cre};*Rbx1*$^{fl/fl}$ mice

As shown, the clusters 1 and 3 shared similar characteristic, while cluster 11 is distinct from clusters 1 and 3.

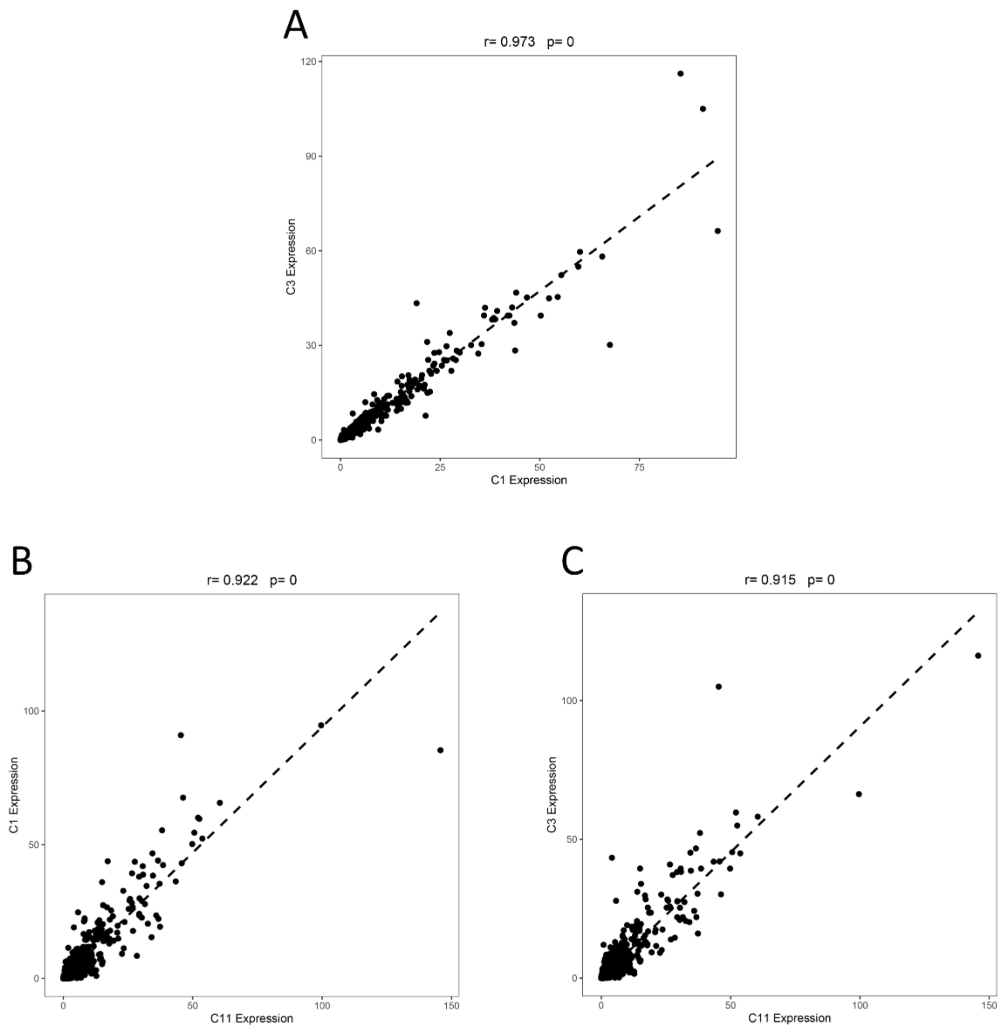

Supplementary Fig. 11. Correlation analysis of cluster 1, 3 and 11 of γδ T cells in thymus from *Lck^{Cre}* and *Lck^{Cre};Rbx1$^{fl/fl}$* mice

A. Cluster 1 v. s. Cluster 3.

B. Cluster 11 v. s. Cluster 1.

C. Cluster 11 v. s. Cluster 3.

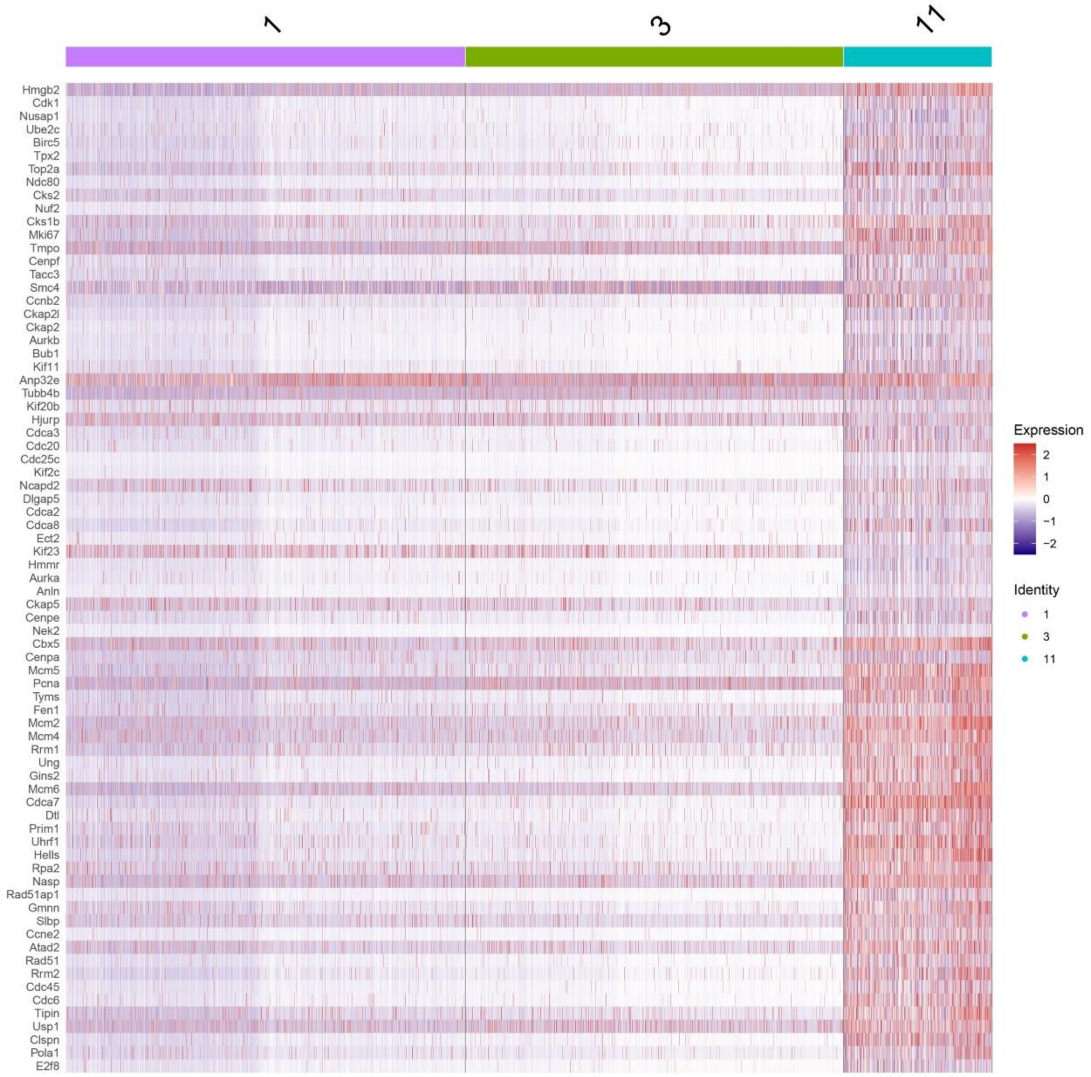

Supplementary Fig. 12. Heatmap of cell cycle analysis of cluster 1, 3, 11 of γδ T cells in thymus from *Lck*^{Cre} and *Lck*^{Cre};*Rbx1*$^{fl/fl}$ mice

Expression patterns of cell cycle related genes among indicated clusters.
